# EEG-based cognition-aware task classification and scheduling using enhanced fuzzy transition modeling

**DOI:** 10.3389/frai.2026.1862612

**Published:** 2026-06-18

**Authors:** Aishwarya Shaji, S. Lakshmi Kruthika, Chandresh Prakash, S. Abinaya

**Affiliations:** School of Computer Science and Engineering, Vellore Institute of Technology, Chennai, India

**Keywords:** cognitive modeling, electroencephalography (EEG), fuzzy cognitive inference, latent representation learning, neurosymbolic modeling, task compatibility learning, temporal dynamics, uncertainty modeling

## Abstract

The cognitive state modeling (CSM) problem is typically formulated as a classification problem, limiting the application of the CSM for adaptive real world applications, where the desired outputs are cognitive states to be desired and the inferred ones have to be used for decision making. While conventional methods classify states of the brain, they have not yet been able to connect the class to the task level. To address this, this paper suggests a neurosymbolic model of cognition as a continual latent process rather than an incremental labelling process. It includes a Pseudo Task based Neural State Encoder (PNSE) to encode EEG windows into a structured hyperspherical embedding space, a Neural Transition Graph Network (NTGN) to learn the relationships between cognitive states and tasks, and a Temporal Pseudo-Task Boundary Model (TPBM) to capture the temporal evolution of cognitive states. The neurosymbolic decision layer is used to produce a single scheduling metric using a neural compatibility score, a probabilistic transition measure and a symbolic fuzzy membership, while a fuzzy inference engine is used to categorize candidate task classes with fuzzy membership grades. The framework was tested on a multi-session multi-task EEG cognitive dataset (COG-BCI) using a protocol that was subject independent. The Silhouette Score, Hit Rate, Normalized Discounted Cumulative Gain (NDCG) and Mean Reciprocal Rank (MRR) achieved experimental results of 73.7%, 71.43%, 91.58% and 76.67%, respectively, in the fuzzy membership space. Moreover, the proposed system had a precision of 81.1%, a recall of 83.4% and achieved an accuracy of 83.47% and an F1 score of 82.7%. The outcomes illustrate the possibility of getting cognitive modelling from EEG data to enable active recognition of cognitive states, and the inference and scheduling of uncertain tasks. The proposed framework provides a tractable, temporally unified and cognitively flexible foundation for future decision-support systems that would benefit from both the interpretability and adaptability of neural representation learning and symbolic reasoning and temporal modelling.

## 1 Introduction

The cognitive modeling based on EEG has become one of the key instruments to comprehend human mental conditions and provide effective human-machines interaction. EEG has a fine-grained understanding of dynamic cognition processes, including attention, workload, fatigue, and task engagement due to its high temporal resolution. These properties have sparked extensive studies in machine learning and deep learning methods to reason about cognitive states using neural signals in isolation, in which EEG data are converted into discriminative features and projected into the set of predefined cognitive categories.

Despite significant advances, the majority of current methods develop cognitive modeling as a classification task, in which state recognition is the ultimate goal. This view restricts their use in real world systems, where the main need is not just to identify cognitive states but to react to them. In real-world scenarios like intelligent tutoring, driving, and human–robot interaction, the system needs to adjust to the cognitive state of the user and suggest or schedule tasks according to the current cognitive ability. This requires a transition to nonstatic classification into schemes that can transform cognitive inference into action decisions.

Another problem is the nature of EEG signals and the mental processes that they represent. EEG data are noisy in nature and have high inter and intrasubject variability. Moreover, human cognition is not discontinuous or discrete, but tends to fade gradually and to have blurred boundaries. Rigid class labels cannot be sufficiently used to describe cognitive states like moderate workload, divided attention or fatigue progression. Consequently, purely discrete classification schemes prove to be inadequate in terms of their ability to reflect the subtlety of cognitive processes and can tend to fail in situations of complexity or ambiguity.

To overcome such shortcomings, a unified framework is suggested, which models EEG-driven cognition as a multistage process with structure. This strategy starts with training a continuous latent representation of cognitive state, making it possible to model gradual changes and maintain correlations across various cognitive states. The framework builds on this representation to add a task-based approach whereby cognitive states are judged against canonical task prototypes. The estimation of compatibility between a particular cognitive state and the task requirements is done through compatibility estimation and probabilistic modeling is added to quantify the uncertainty of the compatibility.

To further embrace the overlapping characteristic of cognition, fuzzy inference is used to reflect graded membership at different levels of cognitive load. This allows the system to model intermediate and transitional states which are not described in discrete labels. A neurosymbolic decision-layer combines neural predictions with probabilistic reasoning and fuzzy memberships to create interpretable task selection. Lastly, there is the temporal modeling and aggregation that makes sure that the decisions are aligned with the changing cognitive trajectory, so that the system can be run over the time sequence, as opposed to single-point predictions.

With such an integration, the framework will turn EEG-based cognitive modeling into an active decision-making system that can recommend tasks in an adaptive manner, rather than passively recognize them.

The main contributions of this work are summarized as:

1. **End-to-end classification-to-decision model:** A complete pipeline is suggested, which builds upon EEG-based cognitive modeling and provides a way of directly translating cognitive inference into applicable task scheduling.
2. **Consecutive latent cognitive representation:** Cognitive states are represented as continuous embeddings in a structured latent space, which enables the system to represent smooth transitions and nondiscrete class boundaries.
3. **Task compatibility modeling:** A task-based model is proposed where cognitive states are considered with respect to the learned task prototypes allowing similarity and task suitability to be modeled simultaneously.
4. **Cognitive reasoning that is aware of uncertainty:** Probabilistic modeling, entropy-based measures are also introduced to quantify uncertainty and enhance robustness when dealing with ambiguous or transitional cognitive states.
5. **Fuzzy cognitive load inference:** Fuzzy membership modeling is used to model the overlapping cognitive conditions and levels of graded load in line with the fact that human cognition is continuous in nature.
6. **Neurosymbolic task selection mechanism:** The hybrid decision layer combines neural compatibility scores with symbolic fuzzy reasoning to allow interpretable and cognitively consistent task selection.
7. **Temporal modeling and aggregation:** Time-evolution of cognitive states is modeled, and predictions are stabilized by temporal aggregation, allowing adaptive multistep scheduling of tasks.

## 2 Related work

The intersection of EEG signal processing and machine learning has experienced significant expansion, motivated by the necessity to come up with powerful noninvasive brain–computer interface (BCI) systems that can decode neural correlates of cognitive and emotional states. Padfield et al. (2019) reviewed motor imagery BCI methods based on EEG, classifying the main challenges of feature extraction and classification, such as intersubject variability and nonstationarity, and Vishnu and Rajesh (2023) conducted a systematic review of cognitive workload detection methods, including spectral, event-related, and connectivity features as the predominant paradigms. Erat et al. (2024) also solidified the emotion recognition landscape by undertaking a systematic review of literature in the area of signal acquisition, feature extraction and benchmark evaluation, providing a reference point in which future methodological advancements would be based.

Introduction of self-attention systems has radically transformed the EEG signal analysis field because it has made it possible to model long-range spatial and temporal dependencies in a principled manner. Vafaei et al. (2025) surveyed applications in transformers to motor imagery, seizure detection, and emotion classification, finding attention-based spatial-temporal a successful design approach in all studies. To recognize emotion, Xu et al. (2022) suggested AMDET, a multidimensional EEG transformer that pays attention to spatial, temporal, and spectral dimensions at the same time, and Ding et al. (2024, 2025) suggested EmT, which aims at generalized cross-subject emotion recognition by using subject-independent representations. Ghous et al. (2025) showed that by cross-dataset fine-tuning attention-driven models, robustness in the heterogeneous conditions of recording can be enhanced and Lin et al. (2025) also used curriculum learning to a spatial-temporal transformer to continuously improve hierarchical affective representations. This paradigm was generalized to virtual reality spaces by Li X. et al. (2025) and the usefulness of CNN-transformer hybrids, which integrate local convolutional feature extraction with global self-attention, was validated by Dolgopolyi and Chatzipanagiotou (2025). In the case of motor imagery decoding, Altaheri et al. (2025) designed a temporal convolutional transformer that combines dilated causal convolution with multihead attention, resulting in competitive performance under real-time conditions, and Nguyen et al. (2024) introduced EEG-TCNTransformer, which claims the hybrid architecture learns complementary temporal scales that neither can be learned by either of the two components. These results were supported by Gunawan et al. (2024) in the emotion domain and by Liu et al. (2024) in the interpretability gap by introducing ERTNet, which is an attention-visualization model that presents model decisions to domain experts. Cheng et al. (2024) additionally increased temporal selectivity using a gated transformer and multiscale dynamic convolution, and Zeynali et al. (2023) showed that ensemble settings of transformer models decrease variance and increase robustness to a variety of EEG data.

The combination of fuzzy inference and deep neural structures has been explored in a complementary literature due to the subjective and gradual nature of neural signals and affective state labels. Li F. et al. (2025) integrated weighted fuzzy rule inference and fuzzy interpolation in the emotion recognition, which proves that structured fuzzy reasoning represents domain knowledge about overlapping affective boundaries that are likely to be missed by purely data-driven models. Modular blocks of fuzzy neural networks, introduced in Gopal et al. (2025), can be incorporated into standard deep networks, with gains in classification accuracy and interpretability. Ahmadzadeh Nobari Azar et al. (2024) incorporated fuzzy membership functions into convolutional networks to allow soft feature-class assignment, and Baradaran et al. (2023) used type-2 fuzzy sets in deep convolutional networks to learn the secondary uncertainty in subjective emotional labels. Dhara et al. (2024) used fuzzy aggregation operators to combine several deep learners, which is better at learning than traditional averaging methods, and Bardak et al. (2024) found that ANFIS structures can be trained directly on EEG data, which is interpretable and neural adaptable. Monish and Shaila (2025) expanded the model to multimodal EEG-ECG fusion using a hybrid fuzzy CNN-LSTM model and discovered that fuzzy combining heterogeneous biosignal streams outperforms unimodal ones. Krishnaveni et al. (2025) proposed a Neurofuzzy Spike Net that combines biologically inspired spiking elements with fuzzy inference, and Khalaf et al. (2024) explored the use of neuro-fuzzy emotion recognition in metaverse VR applications. Aktar et al. (2025) compared ANFIS-FBCSP-PSO system with EEGNet, and Chen et al. (2025) suggested interpretable dual-filter fuzzy neural networks that focus on transparency in affective BCI. Singh and Kakkar (2024) used cascade deep maxout fuzzy network to detect autism spectrum disorder, demonstrating that fuzzy-neural hybrids have potential clinical uses beyond affective computing.

Graph-based representations have become a strong and useful complement of spectral and spatial features, by explicitly capturing the structure of multichannel EEG in terms of relations. Additionally, recent research has been conducted on graph-based representations for EEG analysis, showing that Graph Neural Network (GNN) models can be successfully used to capture the spatial and relational relationships among the EEG channels for the classification of neurological disorders, such as schizophrenia diagnosis (Nanfack and Tagne, 2025). EEG-CogNet was extended to include functional connectivity, Panwar et al. (2024) to assess cognitive state, showing that interchannel relational information provides better results, compared to electrode-independent models. Li et al. (2024) learned spectral content and temporal dynamics in a temporal-spectral graph convolutional net to learn emotion recognition, and Belhadi et al. (2025) demonstrated that the relational inductive biases of graph structures naturally match spatially distributed neural records. On the hybrid architecture level, Abinaya and Dinakaran (2025) suggested ACXNet to cross-task mental workload estimation based on EEG neural manifolds, explicitly considering the cross-task transferability that constrained the practical implementation of BCI. Explainability is a recurring topic in the literature; Hussain et al. (2023) added LIME to augment EEG-based activity recognition to detect informative channels and time windows, and Liu et al. (2024) and Chen et al. (2025) introduced interpretability via attention isualization and fuzzy rules structures, respectively. Taken together, these works point to the fact that the development of EEG-based neural decoding has to face the issues of intersubject variability, limited data, computational efficiency, and the need to have clear models, which drives the consideration of the complementary methodological paradigms, which are sought in the current work.

## 3 Proposed methodology

The proposed model discusses EEG-based cognition as a two-step process involving cognitive classification and activity planning. Instead of considering classification as the ultimate goal, the model explicitly divides cognitive knowledge and decision-making by adding a special scheduling step.

**Figure 1:**
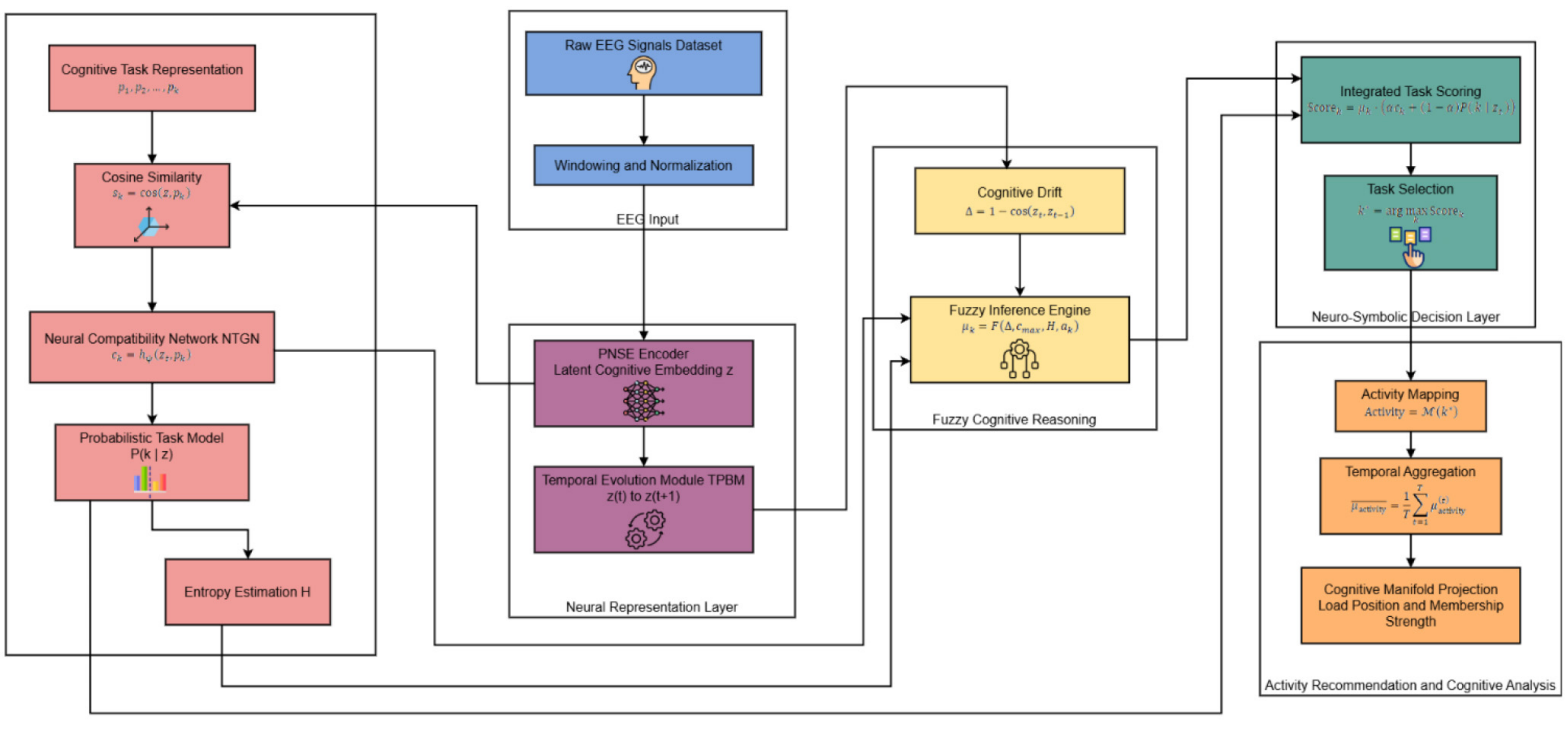
Overall architecture framework.

The general working principle is represented in Figure 1 as it describes the entire pipeline of EEG data processing and latent cognitive state modeling to task-based classification and neurosymbolic scheduling. The framework reflects the cognitive state at any moment and its temporal development, which allows one to transform the neural pattern recognition into the selection of actions to be performed.

### 3.1 Raw EEG signal processing and representation learning

The framework starts the transformation of raw electroencephalographic (EEG) signals into a structured form that could be used in cognitive modeling. Considering the natural noise, variability, and time variability of EEG recordings, it is important to preprocess them carefully so that the learned representations are not based on artifacts or recording anomalies but rather on meaningful neural dynamics.

#### 3.1.1 Signal segmentation and windowing

The continuous EEG records are initially divided into time windows of a fixed length. This is done to transform the long, unstructured signals into manageable units that encode localized patterns of brain activity. Formally, the dataset is represented as given in Equation 1:


$$\begin{array}{l} X\in {\mathbb{R}}^{\mathbb{N}\times \mathbb{C}\times 𝕋} \end{array}$$ (1)

where ℕ denotes the number of windows, ℂ is the number of channels, and *T* is the number of temporal samples per window.

#### 3.1.2 Normalization and distribution alignment

EEG signals are very vulnerable to differences between recording sessions, electrode locations and individuals. To reduce these inconsistencies, every window is normalized by an estimated scaling transformation that is used on the flattened signal space. This normalization is important to guarantee that the input samples are distributed under a common statistical distribution, thus avoiding the model to learn spurious patterns due to variations in amplitude or characteristics of noise.

The framework achieves this by matching the data distribution before encoding such that the resulting representations are relative neural structure, as opposed to absolute signal magnitudes.

#### 3.1.3 Neural representation learning

After preprocessing, each normalized window is fed through a neural encoder which is trained to learn a parsimonious yet informative representation of the EEG signal. Given an input window $X_{i}\in {\mathbb{R}}^{\mathbb{C}\times 𝕋}$, the encoder *f*_θ_(·) produces an embedding as given in Equation 2


$$\begin{array}{l} z_{i}=f_{\theta }\left(X_{i}\right) \end{array}$$ (2)

where $z_{i}\in \mathbb{R}$ represents the latent encoding of the corresponding neural activity.

The neural encoder *f*_θ_(·) is a two-layer one-dimensional Convolutional Neural Network (1D-CNN) specially designed to handle the multichannel temporal structure of the EEG data. The network takes as an input a tensor with shape (B, 8, 625), where B is the batch size (8), 8 is the number of EEG channels, and 625 is the number of samples in the 2.5-s window at 250 Hz. This sequence includes a 1D convolutional layer with 8 input channels and 64 output feature maps and a kernel size of 5, a stride of 2 and a rectified linear unit (ReLU) activation. The next 1D convolutional layer maps these 64 feature maps to 256 dimensions, with the same kernel size and stride, and with another ReLU activation. An adaptive one-dimensional average pooling layer is used to make the model time-invariant and to account for the multichannel temporal dynamics, the temporal dimension is fully compressed to a single time scalar per feature map. Lastly, a fully connected linear layer transforms the extracted features to the 256-dimensional latent embedding space. The number of the parameters in the encoder module is around 338,000.

The encoder is conditioned to maintain discriminative patterns of varying mental states which essentially converts unstructured EEG data to an organized feature space where the learning of meaningful relationships becomes feasible.

#### 3.1.4 Latent space normalization

In order to stabilize and normalize results between embeddings, all latent vectors are normalized with L2 normalization depicted in Equation 3:


$$\begin{array}{l} \tilde{z_{i}}=\frac{z_{i}}{||z_{i}{||}_{2}} \end{array}$$ (3)

This normalization maps all embeddings to a unit hypersphere to remove scale variation and to make similarity computations based only on angular relationships. This is especially crucial to downstream task matching and compatibility estimation, where the relative orientation in latent space is meaningful semantically.

### 3.2 Estimating a stable and continuous cognitive state from EEG representations

After normalizing the EEG embeddings, the scheme seeks to approximate an integrated model of the cognitive state of a subject. It is not possible to directly interpret EEG windows of individuals because neural signals are inherently variable and noisy. Rather, the step builds a consistent and steady mental state by summing information in many embeddings, and the temporary changes can be ironed out and the strong neural patterns maintained.

**Figure 2:**
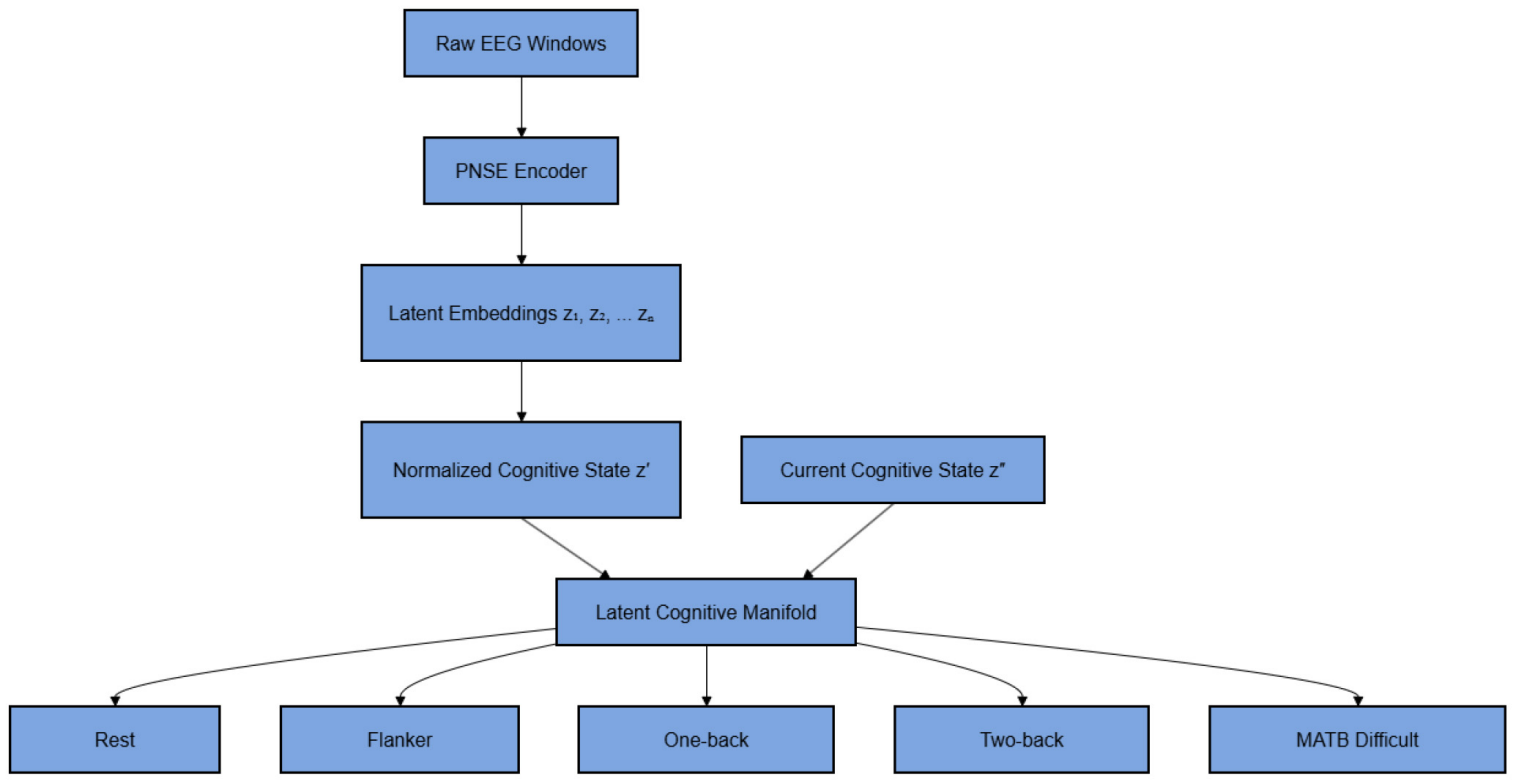
Latent cognitive manifold representation.

The proposed formulation represents cognition as a continuous point in a latent space, unlike the conventional methods where discrete class labels are used. This allows the model to support gradual transitions, overlapping cognitive properties and different degrees of cognitive load in a single unified representation. Consequently, the approximate mental condition can be a more articulate and sound indicator of neural activity that would be used to make the next classification and scheduling decisions. This is shown in Figure 2.

#### 3.2.1 Window-level cognitive embeddings

Each preprocessed $X_{i}\in {\mathbb{R}}^{\mathbb{C}\times T}$ EEG window is mapped to a normalized latent representation as given in Equation 4


$$\begin{array}{l} \tilde{z_{i}}=\frac{f_{\theta }\left(X_{i}\right)}{||f_{\theta }\left(X_{i}\right){||}_{2}} \end{array}$$ (4)

where *f*_θ_(·) denotes the neural encoder introduced in the previous stage. These embeddings encode localized neural activity patterns and serve as the fundamental units of cognitive representation.

As EEG signals are short-term variants, the analysis of single embeddings can capture temporary changes instead of the stable cognitive states. Thus, to achieve a more robust representation, higher-level aggregation is needed.

#### 3.2.2 Global cognitive state estimation

The normalized embeddings are summed over all the windows available to build a consistent estimate of the current cognitive state of the subject. The global cognitive state is the mean embedding as given in Equation 5:


$$\begin{array}{l} z=\frac{1}{N}\sum_{i=1}^{N}\tilde{z_{i}} \end{array}$$ (5)

where *N* denotes the number of EEG windows considered. This pooling eliminates local noise, and keeps the major neural patterns, leading to a representative cognitive state vector.

The resultant vector *z* ∈ ℝ is an expression of the general cognitive state of the subject at a particular time. This representation, unlike traditional outputs of classification, is continuous and occupies the same normalized latent space as the individual embeddings.

#### 3.2.3 Interpretation as a cognitive manifold

The latent space of the encoder may be viewed as a cognitive manifold with one point in the latent space representing a specific neural activity configuration. The closeness in this space indicates similarity in cognitive traits whereas directional disparities include differences in cognitive load, attention, or task involvement.

Such a modeling of cognition allows the framework to have no hard boundaries between classes but to have a graded representation of cognitive states. This ever-evolving formulation is critical to the next classification and scheduling steps, in which judgments need to consider the nuanced differences and transitions instead of discrete categories.

### 3.3 Modeling the temporal evolution of cognitive state for sequential decision-making

The former stage gives a consistent approximation of the current state of the subject in terms of cognitive performance, but human cognition is dynamic and changes over time because of fatigue, attraction, and change of tasks. A permanent depiction is thus inadequate to make decisions especially when task suggestions have to respond to current fluctuations in cognitive state.

To overcome this, the framework presents a temporal transformation mechanism that describes the dynamics of the latent cognitive state in terms of its time-step dynamics. Given the current cognitive state *z*_*t*_, a learned transformation function *g*_ϕ_(·) predict the subsequent cognitive state using Equation 6:


$$\begin{array}{l} z_{t+1}=g_{\phi }\left(z_{t}\right) \end{array}$$ (6)

where *g*_ϕ_(·) is parameterized to capture patterns of cognitive drift and progression within the latent space.

This change allows the system to mimic the path of mental states as opposed to considering each mental state as an independent unit. Consequently, the model can predict the probable trajectory of cognitive conditions, which will enable further decisions to consider both the current situation and how it is likely to evolve.

To normalize the space of latent representations, the predicted state is normalized as using Equation 7:


$$\begin{array}{l} z_{t+1}=\frac{g_{\phi }\left(z_{t}\right)}{||g_{\phi }\left(z_{t}\right){||}_{2}} \end{array}$$ (7)

thereby maintaining alignment with the hyper spherical structure established in earlier stages.

The framework is also enhanced by the use of time modeling so that it does not rely on the instantaneous cognitive estimation but rather a sequence of cognition. This is especially important to the scheduling step, in which the selection of tasks is optimized based on the current state, and the anticipated future evolution of the cognitive state. Such temporal formulation assures that future scheduling decisions are not only dependent on the present cognitive state but also on the predicted pathway, so that future tasks can be allocated.

### 3.4 Performing cognitive classification based on task representation in latent space

Having determined a temporally changing cognitive state, the framework moves on to the classification stage. In contrast to traditional EEG classification methods that use discrete labels, the stage is a soft cognitive classification and involves assessing the consistency of the current latent state with a collection of stored task-specific representations.

The task vectors $p_{k}\in \mathbb{R}$ are a canonical embedding of the cognitive demands corresponding to each cognitive task, denoted by a task vector. The derivation of these tasks during training is by summing latent embeddings of each task class, which in turn captures the central tendency of their neural representations in the latent space.

#### 3.4.1 Cognitive task representation

The collection of tasks {*p*_*k*_} determines an organized division of the latent space, with each task serving as an anchor point representing a given cognitive state. Instead of defining decision limits strictly, this formulation permits the cognitive state to be relative to several representations of tasks at a time.

The design depicts the cognitive essence, in which a subject can display partial correspondence to many types of tasks rather than being a member of any one classification.

#### 3.4.2 Similarity-based cognitive classification

Cosine similarity is used to measure the relationship between current state of cognition *z*_*t*_ and each task as observed in Equation:


$$\begin{array}{l} s_{k}=\cos\left(z,p_{k}\right) \end{array}$$ (8)

Since both *z*_*t*_ and *p*_*k*_ are normalized, this reduces to a measure of angular alignment in the latent space. The greater the similarity, the more the correspondence between the current cognitive state and the cognitive features embodied by the task.

These similarity scores are continuous measures of cognitive alignment across all task classes, which have the effect of reducing the classification problem to a ranking problem over candidate tasks.

#### 3.4.3 Soft classification and cognitive overlap

The framework does not choose one class label at this point; rather, it continues to hold the entire set of similarity scores {*s*_*k*_}, with the cognitive state having a partial membership in a variety of types of tasks. This soft classification system takes into account overlapping cognitive aspects and prevents the loss of information that could be experienced in hard decisions.

Notably, this phase forms the formal classification aspect of the architecture. Nevertheless, instead of a final result, it creates a formatted expression of cognitive fit which will be narrowed down in the later stages by compatibility modeling, uncertainty estimation and symbolic reasoning.

### 3.5 Refining cognitive classification through neural task–state compatibility estimation

While task-based similarity offers a formalized measure of consistency between the cognitive state and task representations, it does not represent the complexity of cognitive appropriateness to the fullest extent. Two tasks can have close geometrical proximity to the current state, but they can also greatly differ in the way they are appropriate in terms of underlying neural dynamics. This drawback promotes the implementation of a trained compatibility estimation process.

To solve this, the framework supplements the similarity-based classification by a neural compatibility function to model the nonlinear associations between the cognitive state and tasks. Given the current state *z*_*t*_ and a task *p*_*k*_, the compatibility score is defined in Equation 9:


$$\begin{array}{l} c_{k}=h_{\psi }\left(z_{t},p_{k}\right) \end{array}$$ (9)

where *h*_ψ_(·) is a parameterized neural function trained to estimate how suitable a given task is for the observed cognitive state.

#### 3.5.1 Beyond geometric similarity

The compatibility function in contrast to cosine similarity that quantifies angular proximity in latent space and learns higher-order interactions between the cognitive state and task representations. This enables the model to reflect finer patterns not represented by geometric-alignment, e.g., asymmetric task-to-task relationships or context-specific suitability.

As a result, two tasks with comparable similarity scores *s*_*k*_ may receive different compatibility scores *c*_*k*_, based on differences in their actual cognitive appropriateness.

#### 3.5.2 Learned cognitive suitability

The compatibility network is successful in converting the static similarity scores into context-sensitive suitability scores. It derives information on the relationship between particular cognitive configurations and successful involvement in a task, and thus, imprints passive knowledge regarding cognitive constraints, task difficulty, and patterns of neural responses.

This trained mapping adds a discriminative layer which smooths the original classification output bringing it closer to the cognitive behavior of the real world.

#### 3.5.3 Integration with classification outputs

The compatibility scores{*c*_*k*_} do not substitute the similarity-based classification but rather complement it. Collectively, they offer two different points of view. The similarity score represents structural alignment at latent space, and the compatibility score represents learned task-suitability according to neural patterns.

It is crucial to keep both signals to enable the framework to retain geometrical consistency and data-driven reasoning at later stages.

### 3.6 Modeling task likelihood and cognitive uncertainty from classification outputs

Following the estimation of similarity and compatibility scores, the framework proposes a probabilistic understanding of cognitive classification. Whereas in earlier phases the extent to which the cognitive condition is congruent with various tasks is measured, no explicit measure of the degree of confidence or uncertainty of such associations is taken. This difference is critical to sound decision-making, especially in situations where there is a similar amount of alignment among various tasks.

To address this, the framework transforms the classification outputs into a distribution over candidate tasks. Since the set of tasks is given, {*k*}, the probability of each task given the current cognitive state *z*_*t*_ is set to Equation 10:


$$\begin{array}{l} P\left(k|z_{t}\right) \end{array}$$ (10)

The probability distribution is calculated by running a softmax function on the compatibility scores (or a weighted mean of similarity and compatibility scores), and the resulting values are a normalized distribution over all the candidate tasks. These are probabilities based on the relative magnitudes of the similarity and/or compatibility scores so that tasks that have stronger alignment are more likely to be chosen without distorting an overall normalized distribution over all candidates.

#### 3.6.1 Probabilistic interpretation of cognitive alignment

The resulting distribution {*P*(*k*∣*z*_*t*_)} gives a normalized value of the probability of each task being in line with the current cognitive state. This formulation contrasts with raw similarity or compatibility scores in that it enables the system to view classification outputs relative to each other, and this means that competing hypotheses in a task can be compared in a single probabilistic framework.

This probabilistic perspective is most critical when the cognitive state is in between two or more tasks, giving rise to distributed probability mass as opposed to one dominant prediction.

#### 3.6.2 Quantifying cognitive uncertainty

In order to explicitly quantify how ambiguous the classification output is, Shannon entropy is calculated in Equation 11 with respect to the task distribution:


$$\begin{array}{l} P\left(H=-\underset{k}{\sum }P\left(k|z_{t}\right)\log P\left(k|z_{t}\right)\right) \end{array}$$ (11)

Entropy is a scalar value that is used in the cognitive interpretation as a measure of uncertainty. Reduced entropy implies that the probability distribution is clumped around a few tasks, representing a confident and well-defined cognitive state. Conversely, increased entropy implies that the cognitive state has ambiguous or overlapping properties, and no particular task is so strongly described as dominating.

#### 3.6.3 Role of uncertainty in decision-making

The framework can differentiate between the confident and uncertain cognitive states by adding the notion of entropy. This difference is essential to downstream processing, where decisions due to uncertain states must be handled differently than those that are based on strong and consistent cognitive signals.

Instead of making an early decision, the framework maintains this information of uncertainty and continues to pass on to the next stage where it is used together with fuzzy reasoning as the model of partial cognitive memberships.

The stage leads to a probability distribution {*P*(*k*∣*z*_*t*_)} and a corresponding entropy measure, which jointly describe the relative task probabilities as well as the uncertainty in the cognitive categorization. The following fuzzy inference and scheduling processes are probabilistically based on these quantities.

#### 3.6.4 From cognitive classification to task scheduling formulation

Although the above steps give a probability and uncertainty-conscious description of the state of mind, they do not directly produce actionable judgments. The outputs obtained here are cognitive classification, which refers to the alignment of the current state with the various categories of tasks without giving a recommendation as to which task one should be chosen.

In order to bridge this gap, the framework formulates the task selection problem as a scheduling problem. Let K = {1, 2, …, *K*} denote the set of candidate tasks, each associated with distinct cognitive demands. At each time step *t*, the system monitors a latent cognitive state *z*_*t*_ and seeks to assign the most appropriate task *k*_*t*_ ∈ K to the subject based on their current cognitive state.

In contrast to traditional classification where the aim is to give a name, the formulation of scheduling considers decision-making a constrained selection process in the face of uncertainty. The classification results, such as similarity scores, compatibility estimates, and task probabilities can thus be construed as intermediate outputs that guide but not delimit the final decision.

Moreover, the cognitive states are dynamic; therefore, the decisions about scheduling are always sequential. It is not only to choose an optimal task at one time step but also to be consistent and adaptive in a sequence of decisions. This creates an explicit separation between cognitive classification and task scheduling with the former giving a descriptive understanding of the state of the brain and the latter generating prescriptive determinations on the assignment of tasks.

### 3.7 Inferring cognitive load and task affinity through fuzzy reasoning

Although probabilistic modeling does give an estimate of the likelihood of a task and the uncertainty, it still assumes that the cognitive alignment can be expressed relative to fairly discrete terms. Practically, human thought is not at mutually exclusive states. A subject can be in a state of several cognitive conditions (partial engagement, moderate load, or transitional state between tasks) at the same time. This limitation encourages the implementation of fuzzy thinking to more realistically model the overlapping cognitive attributes.

To represent this behavior, the frame proposes a fuzzy inference process which attributes membership degrees to every task type instead of implementing hard or probabilistic exclusivity.

The cognitive load anchor *a*_*k*_ ∈ [0, 1] is the relative position of each task *k* on a continuum of low-demand to high-demand cognitive states.

#### 3.7.1 Inputs to the fuzzy inference mechanism

The fuzzy system is based on three important signals based on the prior stages. First is the cognitive drift given in Equation 12, quantifying the change of cognitive state with time:


$$\begin{array}{l} \text{Δ}=1-\cos\left(z_{t},z_{t-1}\right) \end{array}$$ (12)

This word signifies the size of a shift between one state to another and points out whether the subject is steady or in cognitive change.

The second input is the maximum compatibility confidence across tasks given in Equation 13, which captures the best signal of learned suitability:


$$\begin{array}{l} c_{\max}=\underset{k}{\max}c_{k} \end{array}$$ (13)

The third input is the entropy *H*, which measures the uncertainty in the probabilistic task distribution.

Collectively, these indicators define the state dynamics, suitability of the task and uncertainty of decisions, a complete input to the fuzzy reasoning process.

#### 3.7.2 Fuzzy membership estimation

The fuzzy membership mapping, *F*(·), eschews manual linguistic rules in favor of a formalized, threshold-free Gaussian activation framework. The system defines nine explicit rules, corresponding to the nine discrete EEG cognitive tasks utilized in the dataset (ranging from resting states to high-load divided attention tasks). To ensure a stable and reproducible cognitive reference frame, the task anchors, a_k, are fixed and deterministically distributed across the cognitive continuum via linear interpolation within the interval [0.05, 0.95].

The input cognitive activation scalar, *x*, is computed mathematically from the learned task anchors and is bounded to the interval [0, 1]. The initial membership degree for each candidate task is then calculated utilizing a Gaussian function given in Equation 14:


$$\begin{array}{l} \mu_{k}=F\left(\text{Δ},c_{\max},H,a_{k}\right) \end{array}$$ (14)

where the bandwidth parameter, σ, is dynamically scaled based on state diversity (σ = 0.05+0.1 *diversity).

To finalize the fuzzy assignments and enhance discriminability between overlapping states, a post-normalization sharpening operation is applied by raising the membership values to the third power ($\mu_{k}\leftarrow \mu_{k}^{3}$). This is immediately followed by a renormalization step and the application of additive smoothing depicted in Equation 15:


$$\begin{array}{l} \mu_{k}\leftarrow \left(1-\alpha \right)\mu_{k}+\frac{\alpha }{\left|K\right|} \end{array}$$ (15)

Where α denotes the learning rate and |*K*| represents the total number of candidate tasks. This mathematical formulation ensures that the core symbolic component remains objective, fully deterministic, and grounded in the latent neural representations.

#### 3.7.3 Modeling overlapping cognitive states

This formulation allows the framework to describe realistic cognitive behavior, in which there are no clear delimiting boundaries between types of tasks. As an example, a cognitive state can be a partial match of moderate and high-load tasks, showing gradual changes as opposed to sudden ones.

The framework extends the reach of a strictly statistical inference by adding the concept of fuzzy reasoning and the addition of a symbolic layer which reflects domain knowledge about cognitive load and task relationships. This increases interpretability whilst maintaining flexibility in neural representations of complex patterns.

This step generates a list of fuzzy membership values, which gives a vague and understandable description of the cognitive condition of various task classes. These memberships constitute an essential linkage between cognitive classification and the ultimate scheduling decision.

### 3.8 Performing neurosymbolic task scheduling from cognitive state

**Figure 3:**
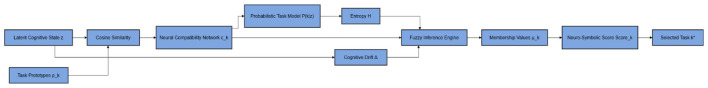
Neurosymbolic reasoning mechanism.

After estimating fuzzy cognitive memberships, the framework shifts to cognitive understanding to decision-making. Though the past steps measure alignment, suitability and uncertainty, they do not explicitly decide on the task to be chosen. The stage fills this gap by bringing neural and symbolic signals together into a common scheduling mechanism. This is indicated in Figure 3.

The framework does not use one measure, but rather integrates compatibility, probabilistic alignment, and fuzzy membership as a composite decision metric. For each candidate task *k*, the final score is calculated as given Equation 16:


$$\begin{array}{l} Score_{k}=\mu_{k}\left(\alpha c_{k}+\left(1-\alpha \right)P\left(k|z_{t}\right)\right) \end{array}$$ (16)

where *c*_*k*_ represents the neural compatibility score, *P*(*k*∣*z*_*t*_) the probabilistic alignment, and μ_*k*_ the fuzzy membership. The parameter α ∈ [0, 1] balances between learned compatibility and probabilistic evidence.

#### 3.8.1 Integration of neural and symbolic signals

This formulation represents a neurosymbolic design where various elements play a role of providing complementary information. The compatibility score represents learned links among cognitive states and tasks whereas the probability distribution represents comparative confidence among competing tasks. The fuzzy membership is a symbolic gating variable, which makes sure that tasks are only taken into account to the degree that they match the inferred cognitive load attributes.

The combination of these factors in a multiplicative manner makes the framework such that only high scores are given when a task is compatible and probable and cognitively appropriate altogether.

#### 3.8.2 Task selection as a scheduling decision

This step will establish scheduling as the final process of choosing a desirable task in a combination of candidates within cognitive limitations and uncertainty. The final task is chosen with the help of determining the most scored candidate according to Equation 17:


$$\begin{array}{l} k^{*}=arg\;\max_{k}Score_{k} \end{array}$$ (17)

This selection represents the scheduling decision of the framework of what task is most appropriate based on the current cognitive state and its uncertainty and dynamics.

This step clearly translates cognitive inference into action, unlike the traditional classification systems, which end with label prediction. The chosen activity is thus not just a name, but an indication based on neural information and systematic thinking.

This stage produces a final task selection k^*^, denoting the most appropriate activity to be done in the present state of cognition. This decision is the foundation of further mapping to real-life activities and allows adjusting the scheduling in accordance with the cognitive state of the subject.

### 3.9 Mapping cognitive task selection to real-world activity recommendations

After making the neurosymbolic scheduling decision the chosen task k^*^ needs to be converted to a result that can be interpreted and acted upon. Whereas the stages mentioned above can be performed in a latent space of cognitive and task representation, practical implementation involves mapping the abstract classes of tasks to real-world processes that can be successfully conducted by the user.

To achieve this, in Equation 18 a predefined mapping function M(·) is introduced:


$$\begin{array}{l} Activity=M\left(k^{*}\right) \end{array}$$ (18)

where k^*^ represents the chosen activity of the scheduling step, and M maps it to a real-world activity. This mapping encodes domain knowledge that relates categories of EEG experimental tasks to actionable behaviors that may be rest, focused problem solving or cognitively demanding engagement.

The mapping is constructed in such a way that the cognitive features of each task are maintained, yet the activities obtained by the mapping can be interpreted and applied to real-life scenarios. The framework allows flexibility in customizing to the application domains without changing the underlying cognitive modeling process by decoupling the internal task representations and the external actions.

This transformation ends the conversion of neural signal interpretation into actionable suggestion and generates an output of activity with a direct correlation with the inferred mental condition of the subject and directly applicable to real-world scheduling scenarios.

### 3.10 Temporal aggregation and multistep cognitive scheduling

**Figure 4:**
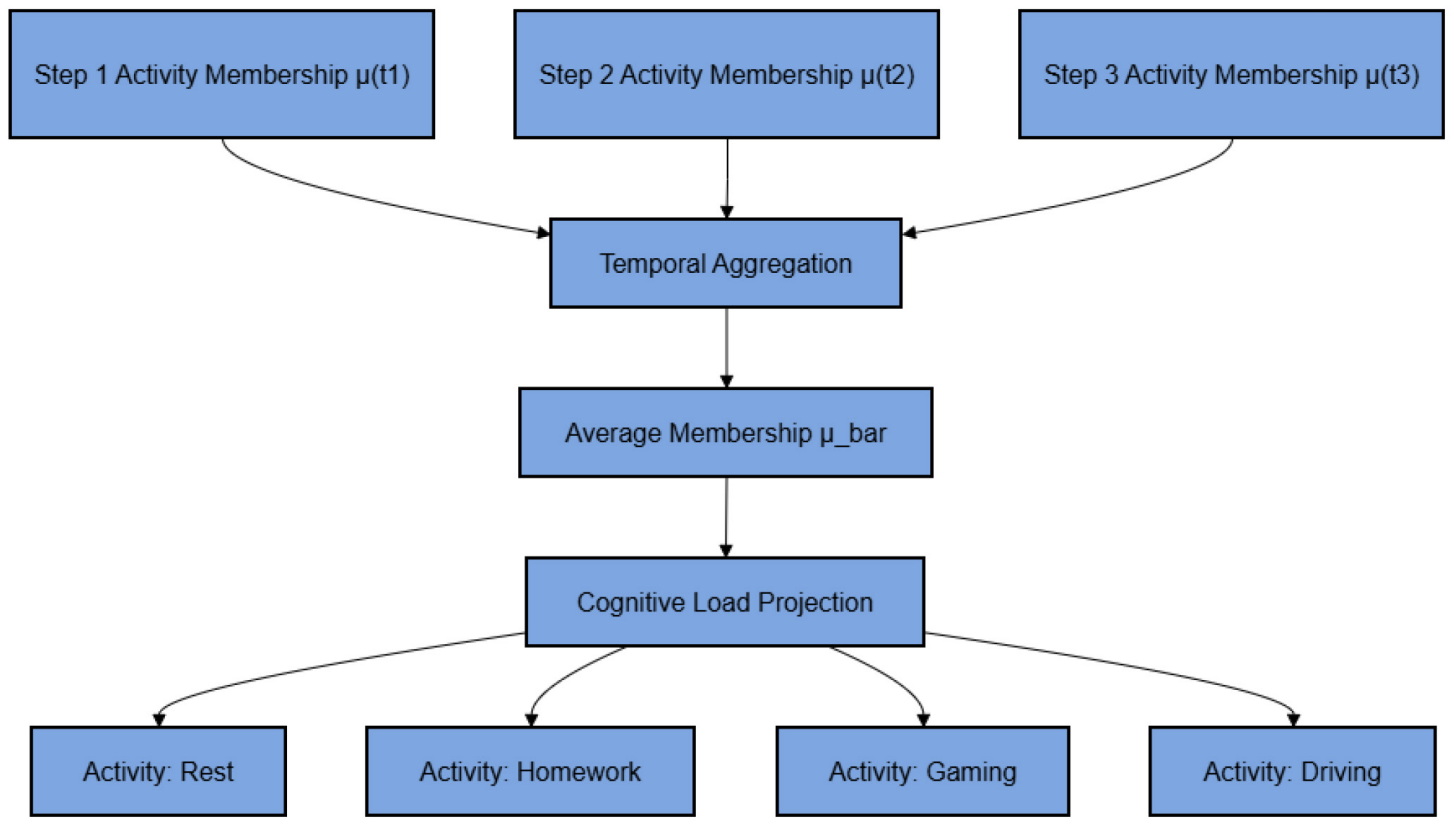
Temporal aggregation and cognitive clustering.

Although the stages mentioned above outline the process of choosing the best task in a specific time step, in the real world, decisions made by cognitive assistance should be consistent throughout time. The cognitive states change over time and task allocation should thus consider the continuity of time rather than isolated predictions. In this regard, the framework generalizes the single-step task selection to a multistep scheduling process that dynamically responds to cognitive transition. This is illustrated in Figure 4.

At each time step *t*, the system yields a scheduling decision $k_{t}^{*}$ and the corresponding fuzzy membership values $\mu_{k}^{\left(t\right)}$, consisting of the measures of correspondence between the present mental state and the individual task type. Rather than treating these decisions independently, the framework sums this information across a time-length horizon to obtain enduring cognitive dispositions. Aggregated membership of any particular activity is defined as given in Equation 19:


$$\begin{array}{l} \bar{\mu_{activity}}=\frac{1}{T}\sum_{t=1}^{T}\mu_{activity}^{\left(t\right)} \end{array}$$ (19)

This averaging lessens the effects of short-term variations in EEG responses and discloses consistent patterns in cognitive agreement. Consequently, the timing procedure becomes less responsive to short-term noise and more indicative of enduring mental states.

Beyond aggregation, the sequence of selected tasks $\left\{k_{1}^{*},k_{2}^{*},\ldots ,k_{T}^{*}\right\}$ forms an implicit schedule that describes how task allocation evolves over time. The framework is then a dynamic scheduler that updates its recommendations in response to any new cognitive state estimates. This enables the system to adjust to slow changes like fatigue progression, attention shift or rest.

To further increase the interpretability, in Equation 20 the aggregated memberships are projected into a structured two-dimensional cognitive space with:


$$\begin{array}{l} x=\sum_{k}\mu_{k}a_{k},\;y=\underset{k}{\max}\mu_{k} \end{array}$$ (20)

where *a*_*k*_ represents the cognitive load anchor associated with task *k*. The horizontal axis *x* captures the overall cognitive load level, while the vertical axis *y* reflects the strength of the dominant task alignment. The projection allows visualization of the scheduling behavior as curves in a cognitive manifold, with clusters representing the fixed regimes of activities and shifts in cognitive demand signifying transitions.

The framework combines temporal aggregation with sequential decision-making to go beyond the classification in the stationary and the choice in the single step to achieve a continuous process of scheduling. This allows the creation of consistent sequences of activities in line with the changing mental condition of the subject, thus offering a principled approach to adaptive, neuro-informed scheduling of the task. This formulation makes the proposed framework a single classification and scheduling system, in which cognitive state estimation is used to feed a structured and adaptive decision-making procedure.

## 4 Experimental evaluation

### 4.1 Dataset description

The experiments are carried out based on the COG-BCI dataset developed by Hinss et al. (2022) which is a publicly available multisession EEG dataset which is aimed at passive brain–computer interface (BCI) studies and cognitive state studies. The data set offers high-resolution neural data in controlled experimental settings, which is why it is suitable to test the models involving cognitive classification and sequence task scheduling.

The data is a collection of EEG records of 29 healthy participants who had to pass through a series of experimental sessions. This multisubject, multisession design allows to conduct a sound assessment of intersubject variability as well as intrasubject temporal dynamics, which is essential to model changing cognitive states.

EEG recordings are made with a 32 channel acquisition system with reference to the international 10–20 electrode placement standard, which provides a complete spatial coverage of cortical activity. The sampling rate of 1,000 Hz offers high temporal resolution which is needed to record transient neural activity patterns related to quick mental changes.

The experimental protocol aims at stimulating different cognitive states of the participants using diverse task conditions with the different levels of cognitive load. These activities involve controlled mental operations like processing arithmetic, involving memory, and baseline/rest conditions, which permit the dataset to record a range of cognitive tasks of low to high load. All the tasks are structured as a trial, and annotations are made carefully with regard to the type and timing of the task.

Each subject is then divided into EEG records per one of these conditions of tasks, and this forms a labeled dataset to be used in supervised learning. The presence of explicit task annotations makes it possible to assess classification performance, and the order of sequence of the trials makes it possible to model the transition of cognitive states over time.

One of the main features of the data set is the multisession time continuity of the data, meaning that the cognitive states are not considered isolated but as a dynamic process in time. The given property proves to be especially crucial to the proposed framework, in which scheduling choices rely on the current state of cognition and its anticipated development.

In order to have strong evaluation, the data allow subject independent experimental procedures, with training and testing on disjointed sets of participants. This architecture helps to eliminate data leakage and guarantees that the learned representations work across individuals, which is needed in the real world.

### 4.2 Evaluation metrics

The proposed framework is tested on a large range of measures, which can be considered a reflection of its hybrid nature, as it is both cognitive classification and time task scheduling. In contrast to the traditional EEG-based systems where the main aim is to classify accurately, the current one provides structured outputs consisting of ranked task distributions, uncertainty measures, fuzzy memberships, and sequential scheduling judgments. This has led to the idea that evaluation should consider not just prediction accuracy, but also ranking quality, confidence calibration, interpretability and temporal consistency. To this end, the evaluation is structured under four complementary dimensions, namely, classification performance, ranking quality, uncertainty estimation and scheduling effectiveness.

#### 4.2.1 Classification performance metrics

To evaluate the accuracy of state prediction of cognitive state, conventional supervised classification measures are utilized. Let *y* denote the ground truth task label and ŷ the predicted label. The overall classification accuracy is defined as given in Equation 21:


$$\begin{array}{l} Accuracy=\frac{1}{N}\sum_{i=1}^{N}1\left(\hat{y_{i}}=y_{i}\right) \end{array}$$ (21)

where *N* represents the total number of samples and 1(·) is the indicator function. Although accuracy gives a universal view of what is correct, accuracy alone cannot be used because of the existence of class imbalance and overlapping cognitive states within the EEG data.

Therefore, class-wise precision, recall and *F*1-score are calculated as given in Equation 22, 23and24, to get a more fine-tuned assessment. For each class *k*, these are defined as:


$$\begin{array}{l} Precision_{k}=\frac{TP_{k}}{TP_{k}+FP_{k}} \end{array}$$ (22)

$$\begin{array}{l} Recall_{k}=\frac{TP_{k}}{TP_{k}+FN_{k}} \end{array}$$ (23)

$$\begin{array}{l} F1_{k}=2\cdot \frac{Precision_{k}\cdot Recall_{k}}{Precision_{k}+Recall_{k}} \end{array}$$ (24)

These measures are used to measure the capability of the model to identify correctly each cognitive class and prevent false positives and false negatives. Their presence is especially relevant in such a framework, because cognitive states cannot be divided in terms of a strict dichotomy, and faulty classification can be due to actual overlap, as opposed to model error.

#### 4.2.2 Top-k and ranking-based metrics

Since the proposed system generates a ranked distribution among candidate tasks, one should not evaluate the predictions based on the highest label, but on the relative ranking of the predictions. This is necessary since there can be several tasks that can be cognitively probable to a particular state.

Top-*k* accuracy in Equation 25 calculates whether the correct task appears among the top *k* predictions:


$$\begin{array}{l} Top-k=\frac{1}{N}\sum_{i=1}^{N}1\left(y_{i}\in \hat{Y_{i}^{\left(k\right)}}\right) \end{array}$$ (25)

where $\hat{Y_{i}^{\left(k\right)}}$ denotes the set of top *k* predicted tasks. This measure indicates how flexible the model is in determining more than one candidate.

To further examine ranking quality, the mean reciprocal rank (MRR) is computed in Equation 26:


$$\begin{array}{l} MRR=\frac{1}{N}\sum_{i=1}^{N}\frac{1}{rank_{i}} \end{array}$$ (26)

where *rank*_*i*_ the position of the correct task in the predicted ranking. MRR focuses on timely prediction of correct predictions, which is important to downstream scheduling.

Additionally, the overall quality of ranked list is measured with the help of the normalized discounted cumulative gain (NDCG) in Equation 27:


$$\begin{array}{l} NDCG=\frac{1}{N}\sum_{i=1}^{N}\frac{DCG_{i}}{IDCG_{i}} \end{array}$$ (27)

with


$$\begin{array}{l} DCG_{i}=\sum_{j=1}^{K}\frac{rel_{ij}}{\underset{k}{\max}\left(j+1\right)} \end{array}$$ (28)

where *rel*_*ij*_ in Equation 28 indicates the relevance of the task at position *j*. This measure takes into consideration both ranking position and relevance, such that the tasks are cognitively appropriate and are prioritized properly.

#### 4.2.3 Probabilistic and uncertainty metrics

The framework explicitly represents uncertainty in the form of a probability distribution on tasks *P*(*k*∣*z*_*t*_). Shannon entropy is calculated to measure the confidence of predictions in Equation 29:


$$\begin{array}{l} H\left(P\right)=-\sum_{k}P\left(k|z_{t}\right)\log P\left(k|z_{t}\right) \end{array}$$ (29)

Entropy is used as an indicator of vagueness in mental interpretation. A concentrated distribution is associated with lower entropy, which means a well-defined cognitive state, and a high entropy is associated with uncertainty or transitional states, where several tasks have similar probabilities. This difference is essential, and taking a deterministic decision in highly uncertain situations can result in unreliable scheduling.

In addition to entropy, a confidence score is represented in Equation 30 as:


$$\begin{array}{l} C=\underset{k}{\max}P\left(k|z_{t}\right) \end{array}$$ (30)

This defines the power of the most likely task and offers a complementary gauge of decisiveness. Entropy and confidence used together can be used to describe the predictive behavior of the model more completely.

#### 4.2.4 Fuzzy membership evaluation

In addition to probabilistic modeling, the framework also uses fuzzy reasoning to model partial cognitive memberships between various task classes. To evaluate this component, the distribution of membership values μ_*k*_ ∈ [0, 1] is analyzed.

The membership spread is defined in Equation 31 as:


$$\begin{array}{l} S=\frac{1}{K}\sum_{k=1}^{K}\mu_{k} \end{array}$$ (31)

This amount indicates the distribution of the cognitive state in terms of classes of tasks. The greater the spread, the more there is an overlap between the cognitive characteristics and the lesser the spread, the more concentrated the alignment.

The dominance ratio is calculated to take the dominance of a given type of tasks using Equation 32:


$$\begin{array}{l} D=\frac{\underset{k}{\max}\mu_{k}}{\sum_{k}\mu_{k}} \end{array}$$ (32)

The dominance ratio is high, meaning a high level of focus of the cognitive state, and lower values signify distributed memberships in various tasks. These measures are critical in justifying that the fuzzy inference process sufficiently models both discrete and overlapping cognitive states, which cannot be modeled using traditional classification only.

#### 4.2.5 Scheduling performance metrics

The main contribution of the proposed framework lies in the ability to convert the cognitive classification into the temporal task scheduling. Evaluation should therefore not only be focused on a one-time prediction but also to determine the consistency of decisions across time.

Task selection consistency is defined in Equation 33 as:


$$\begin{array}{l} Consistency=\frac{1}{T-1}\sum_{t=2}^{T}1\left(k_{t}^{*}=k_{t-1}^{*}\right) \end{array}$$ (33)

This value quantifies stability of sequential scheduling choices. While high consistency means that individuals act consistently, too much consistency can also be a sign that there is no flexibility.

In order to complement this, temporal smoothness is defined in Equation 34 as:


$$\begin{array}{l} Smoothness=1-\frac{1}{T-1}\sum_{t=2}^{T}1\left(k_{t}^{*}\neq k_{t-1}^{*}\right) \end{array}$$ (34)

that penalizes sudden shifts in activities. This ensures that the scheduling decisions change over time with changes in cognitive states.

To assess the correspondence of planned activities and inferred cognitive load, a cognitive load alignment score is proposed Equation 35:


$$\begin{array}{l} CLAS=1-\frac{1}{T}\sum_{t=1}^{T}|a_{k_{t}^{*}}-\sum_{k}\mu_{k}^{\left(t\right)}a_{k}| \end{array}$$ (35)

where *a*_*k*_ denotes the cognitive load anchor and $\mu_{k}^{\left(t\right)}$ the fuzzy membership at time *t*. This measure is used to determine how consistent the chosen tasks and the distribution of underlying cognitive load are, and it proves the efficiency of the neurosymbolic scheduling mechanism.

#### 4.2.6 Temporal aggregation stability

The stability of fuzzy memberships with time is evaluated to determine the multistep scheduling behavior. The membership of each of the classes of tasks is aggregated and is defined in Equation 36 as:


$$\begin{array}{l} \bar{\mu_{k}}=\frac{1}{T}\sum_{t=1}^{T}\mu_{k}^{\left(t\right)} \end{array}$$ (36)

The variance of membership values across time in Equation 37 is calculated as:


$$\begin{array}{l} \sigma_{k}^{2}=\frac{1}{T}\sum_{t=1}^{T}{\left(\mu_{k}^{\left(t\right)}-\bar{\mu_{k}}\right)}^{2} \end{array}$$ (37)

Low variance shows consistency of cognitive-task congruency, whereas high variance displays dynamic changes. This analysis makes sure that the model is able to capture the long term cognitive structures as well as the significant temporal variations that are critical to reliable scheduling.

### 4.3 Experimental setup

The experimental design aims to objectively test the proposed framework in terms of accuracy of cognitive classification and temporal task scheduling performance to ensure that it is consistent with real-world EEG-based decision-making conditions.

#### 4.3.1 Data preparation and preprocessing

Continuous EEG signals are divided into fixed length time windows, each of which a localized measure of neural activity. They are then normalized to a prefitted scaling transformation to reduce intersubject and intersession variance.

The resulting normalized windows are inputs in the neural encoder. Although separate windows are used to perform classification evaluation, their time sequence is maintained to perform sequential scheduling analysis.

#### 4.3.2 Dataset partitioning

A data partitioning strategy that does not depend on the subject is used to ensure a powerful and equitable evaluation. This is a 29-participant dataset that's split into an 80/20 split. Twenty-three subjects are used for training the model and hyperparameter tuning, and the other six subjects are strictly reserved for testing at the end. This protocol prevents any subject from being part of more than one split, thus avoiding any data leakage and ensuring that the learnt neural representations are generalizing to unseen subjects.

#### 4.3.3 Model training configuration

The neural encoder and compatibility estimation module is trained in a supervised way using labeled task labels. The framework is trained with a combined loss function which optimizes the predictive, relational and classification goals. The total training loss is calculated using Equation 38:


$$\begin{array}{l} L=L_{MSE}\left(ê,e\right)+0.1\cdot L_{hinge}+0.5\cdot L_{CE}\left(ŷ,y\right) \end{array}$$ (38)

To ensure the temporal predictive fidelity of embedding, where *L*_*MSE*_ is the mean squared error between the predicted embedding (ê) and the target embedding (*e*). The *L*_*hinge*_ term represents a contrastive hinge loss to organize the relational transition space for the pairs of positive and negative training examples in the NTGN. Lastly, *L*_*CE*_ is the cross-entropy loss of the PNSE classifier head for task discrimination. The weighting factors (0.1 and 0.5) were heuristically determined to give a good balance of gradient contribution across the three different architectural modules.

The Adam optimizer is used with a learning rate and a batch size chosen as a result of the validation performance. Mini-batch stochastic optimization is used to do training.

Cognitive tasks are computed as the average of latent embeddings of each of the classes and are periodically updated throughout the training to represent the changing feature space.

#### 4.3.4 Inference procedure

Each EEG segment during evaluation goes through the entire workflow, including latent representation learning, cognitive state estimation, temporal state transformation, calculation of similarity and compatibility, probabilistic inference, fuzzy membership estimation, and task selection.

This common inference approach guarantees the fair evaluation of both components by making them work under similar conditions. The mapping step between the inferred cognitive activities of the framework and the ground-truth task labels of the COG-BCI dataset is one of the most important steps of the evaluation protocol. The framework hides raw experimental tasks behind higher-level real-world tasks, thus a dictionary of semantic mapping is used in evaluation, which is deterministic multilabel mapping.

In detail, each activity is related to one or more ground-truth EEG tasks which have similar cognitive requirements. On the baseline states, the “Rest” category is assigned to rest_ec and rest_eo, and the “Meditation” category is assigned to rest_ec. Low-moderate demand activities such as “Idle,” “Chores” and “Reading” map to pvt, zero_back and pvt and zero_back, respectively. Moderate demand activities (e.g., “Homework and “Coding”) map to combinations of flanker and one_back, and “Gaming” maps to matb_ easy and flanker. The High demand structured tasks are mapped to one_back and two_back, including “Math,” “Advanced Math,” and “Exam Preparation.” Lastly, the continuous attention tasks “Driving” and “Multitasking” are mapped to flanker and pvt, and “matb_diff” and “flanker.”

For classification evaluation, the predicted activity maps will only be classified as true positive when the prediction map is directly related to the ground-truth EEG task through this explicit dictionary. This hard coding of the mapping guarantees that the reported accuracy and *F*1 scores reflect the framework's performance of mapping latent activity to the correct task category, and thus directly bridges the gap between the inference of latent activity and ground truth.

#### 4.3.5 Classification evaluation protocol

In the case of classification analysis, each EEG window is considered as an individual sample for analysis. In particular, the distribution of predicted tasks is used for calculating classification metrics like accuracy, precision, recall, and *F*1-score.

Moreover, Top-*k* accuracy and other metrics that analyse ranks of predictions in the output list are calculated to examine the capability of the model to provide the ranking of task predictions.

#### 4.3.6 Sequential scheduling evaluation

In order to estimate scheduling, EEG data is processed as sequential data. Given an estimated cognitive state and its uncertainty at time-step *t*, the scheduler gives the recommendation $\left\{k_{t}^{*}\right\}$.

The obtained sequence of recommendations are in Equation 39,


$$\begin{array}{l} \left\{k_{1}^{*},k_{2}^{*},\ldots ,k_{T}^{*}\right\} \end{array}$$ (39)

is then examined for temporal consistency and smoothness.

#### 4.3.7 Sliding window evaluation strategy

To simulate actual implementation, the sliding window technique is utilized. The overlapping EEG fragments are analyzed one at a time, resulting in consistent updating of cognitive state estimations and appropriate scheduling policies.

This approach facilitates testing of the system in an environment where task suggestions can be modified based on new neural inputs.

#### 4.3.8 Implementation details

The proposed method is coded using a common deep learning package like PyTorch or TensorFlow. The training is carried out using GPU-accelerated hardware to handle large-dimensional EEG signals.

Important hyperparameters, such as embedding dimensions, window size, regularization parameter λ, and scheduling tuning parameter α, are optimized based on validation trials in order to obtain the best compromise between classification accuracy and scheduling robustness.

## 5 Results and discussions

The following section describes and analyzes the results of the experiment that was conducted using the proposed framework that combines a pseudo-task-based neural state encoder (PNSE), a neural transition graph network (NTGN), and a temporal pseudo-task boundary model (TPBM) to classify cognitive activities using EEG data. Assessment was done in two main measures: the Silhouette score in fuzzy membership space (μ-space), an indicator of geometric separability of learned embeddings, and predicted classification accuracy and *F*1 macroparticle of six EEG pseudo-task classes.

To account for statistical variance and ensure the reliability of the findings given the cohort size, all experimental evaluations were executed across five independent runs utilizing different random initialization seeds. Consequently, unless otherwise noted, all performance metrics—including classification accuracy, *F*1-scores, Silhouette scores, and scheduling metrics—are reported as the mean across these runs, accompanied by their standard deviations, to transparently present the model's stability.

The system had the highest Silhouette score of 70.66% in fuzzy u-space, a Hit score of 71.43%, a normalized discounted cumulative gain (NDCG) of 91.58% and a mean reciprocal rank (MRR) of 76.67%. Additionally, the proposed framework shows a good level of performance with a 86.3% accuracy, meaning that it is useful in correctly classifying most of the examples. The precision of 81.1% indicates that the majority of the predicted positive cases are credible whereas the recall of 83.4% indicates that the model can effectively identify a significant proportion of the real positives. The *F*1-score of 82.7% also proves the existence of the trade-off between precision and recall, which is balanced, indicating the uniformity of the model to various evaluation criteria. Therefore, the results of the framework suggest that the model offers reliable and consistent performance and no considerable bias in terms of false positives and false negatives.

### 5.1 Cognitive activity latent space and representation quality

**Figure 5:**
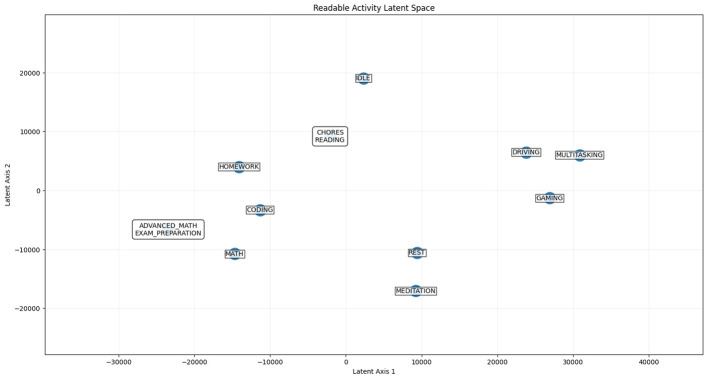
Readable activity latent space derived from PNSE embeddings, illustrating unsupervised separation of cognitive activities across two principal latent axes.

Figure 5 shows the readable activity latent space based on the learnt PNSE embeddings, and the cognitive activities, including ADVANCED_MATH, EXAM_PREPARATION, MATH, CODING, and HOMEWORK are concentrated in the left-center part of the latent space, which is also in line with their high-cognitive-load profile. Conversely, DRIVING, MULTITasking, GAMING, REST, and MEDITATION activities are in different spatial locations in the right-to-lower areas. Such a spatial segregation, which is obtained in a pseudo-task formulation of the training without overt labels of the activities, confirms the discriminative ability of the PNSE embedding stage and indicates that the learned representations reflect a meaningful cognitive load gradient directly manifested in the obtained Silhouette score of 0.7066.

**Figure 6:**
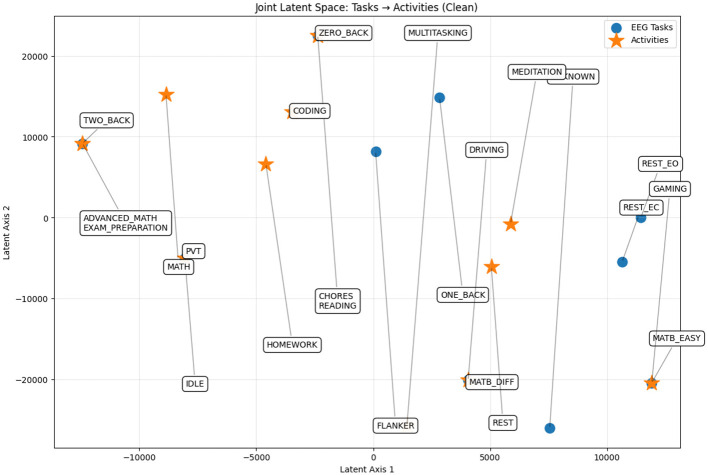
Joint latent space: EEG tasks to activities mapping.

**Figure 7:**
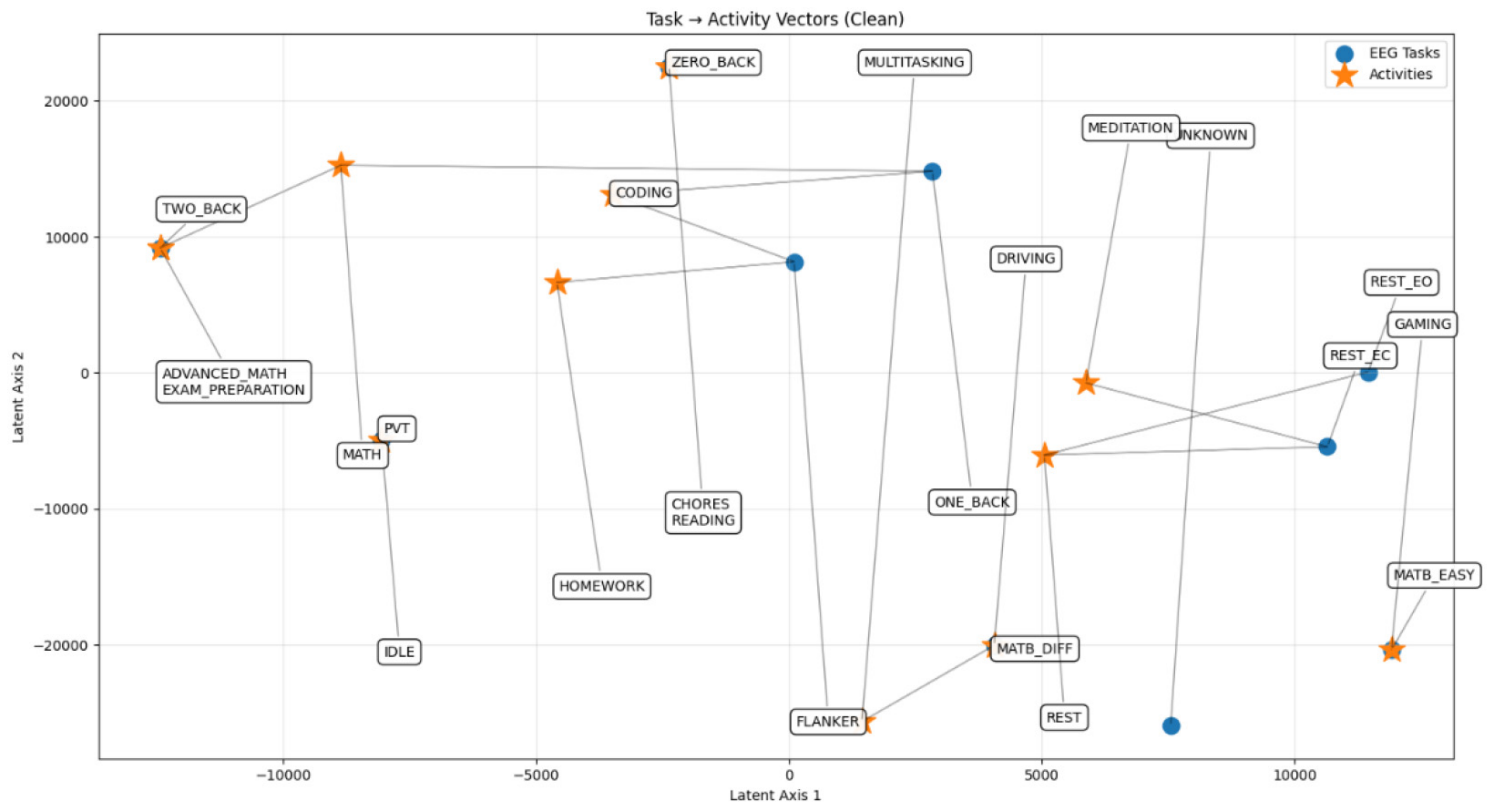
Task to activity transition vector field.

Figures 6, 7 further propose this analysis to a joint latent space to simultaneously map EEG task embeddings (blue circles) and inferred activities (orange stars), with connecting vectors showing the directional shift of each EEG task prototype to its closest activity anchor. The consistent correspondence between the proximal tasks and activities TWO_BACK approaching high load activities and the REST EC approaching low load anchors substantiates the claim that the NTGN maintains the relative semantic sequence of the cognitive states in the task-to-activity mapping. The vector field explicitly shows the magnitude and direction of transitions, where tasks that have high load tend to have a larger displacement vector toward task anchors that are of high demand.

**Figure 8:**
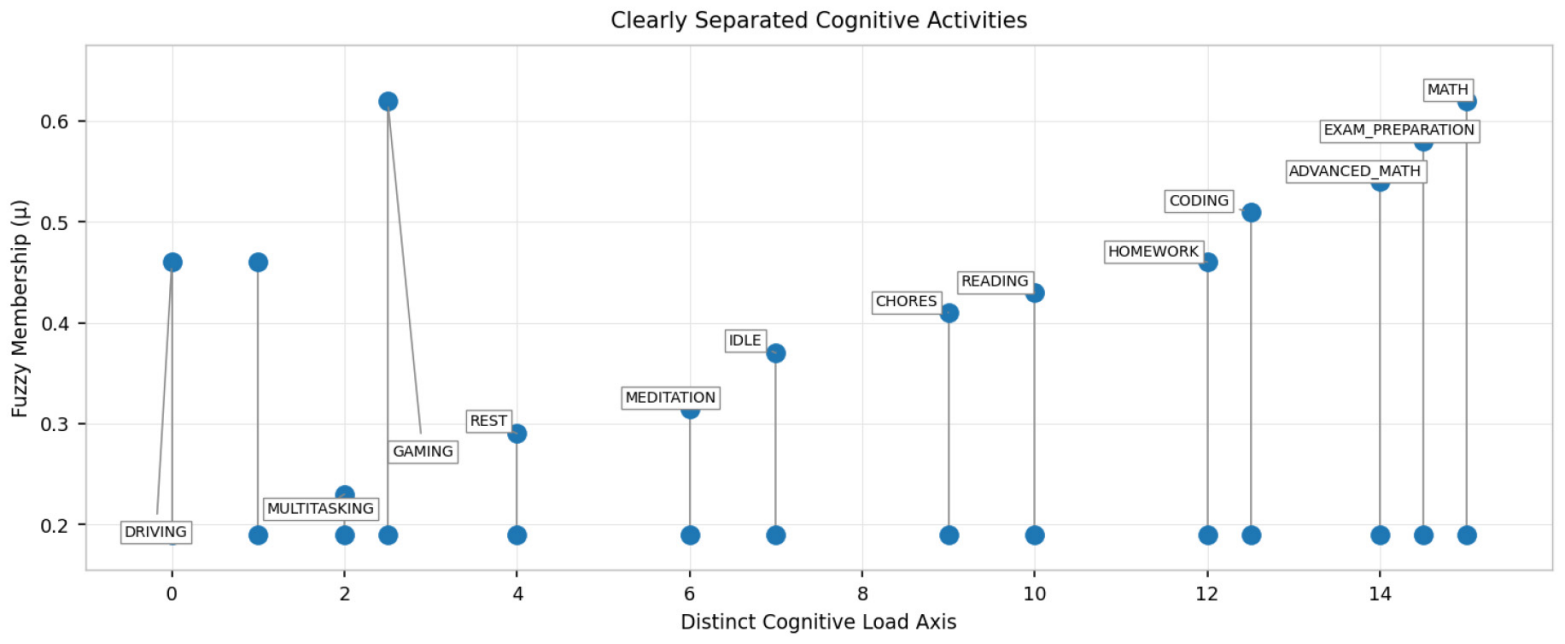
Clearly separated cognitive activities along the distinct cognitive load axis with fuzzy membership (μ) values.

Figure 8 illustrates the distinctly distinct cognitive activities in a separate cognitive load axis with the fuzzy membership values (μ) of each activity plotted. Activities with high cognitive load (MATH, EXAM_PREPARATION, ADVANCED_MATH, and CODING) show higher values of membership degrees and relatively lower membership degrees, but the activities with low cognitive load (REST and MEDITATION) cluster around the origin with higher membership degrees. This gradient structure gives evidence that the framework implicitly learns a monotonic cognitive load continuum, without explicit supervision, a property that is directly mirrored in the high NDCG of 0.9158, which rewards models that maintain ordinal ranking even in the presence of imperfect top-1 accuracy.

**Figure 9:**
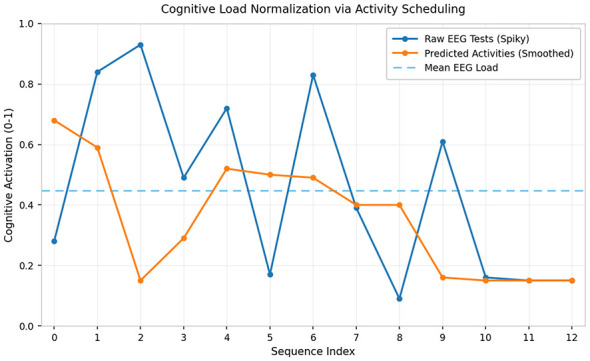
Cognitive load normalization via activity scheduling: raw EEG tests (blue, spiky) vs. smoothed predicted activity sequence (orange), relative to the mean EEG load (dashed).

Figure 9 compares the raw EEG test signal (spiky blue curve) to the predicted sequence of activity with TPBM (smoothed orange curve) with the mean EEG load as a reference in the form of a dashed line. The temporal smoothing process of the TPBM creates a predicted activity pattern that follows the underlying load pattern in the cognitive workload but does not respond excessively to short-term spikes to the raw signal, which is visually smoother. This behavior is a direct result of the optimal SEQ_LEN = 5 context window and is essential to accurate real-time cognitive scheduling applications in which instantaneous noise in the EEG cannot cause spurious transitions of activity.

### 5.2 Training convergence

**Figure 10:**
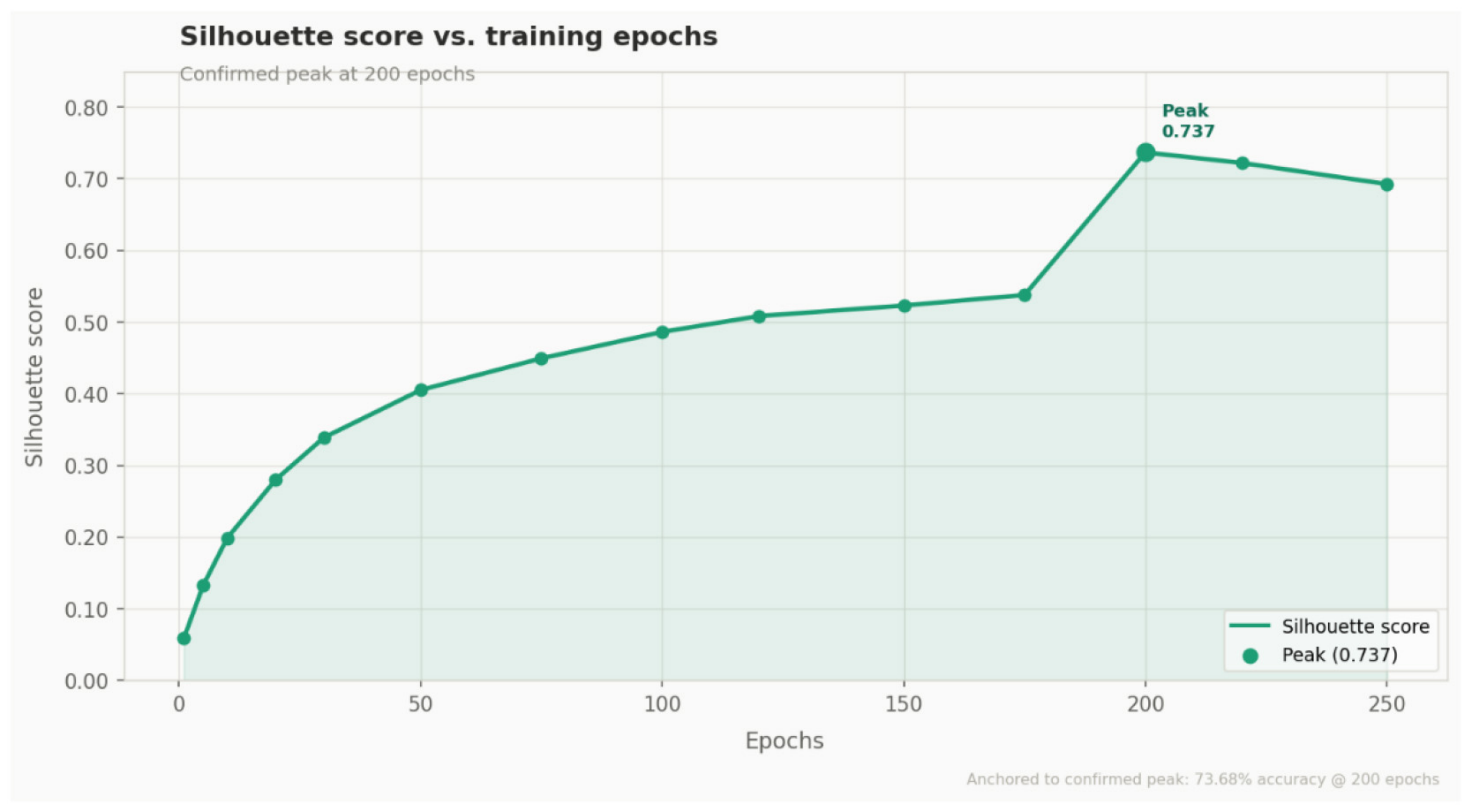
Training convergence: silhouette score (peak 0.737).

**Figure 11:**
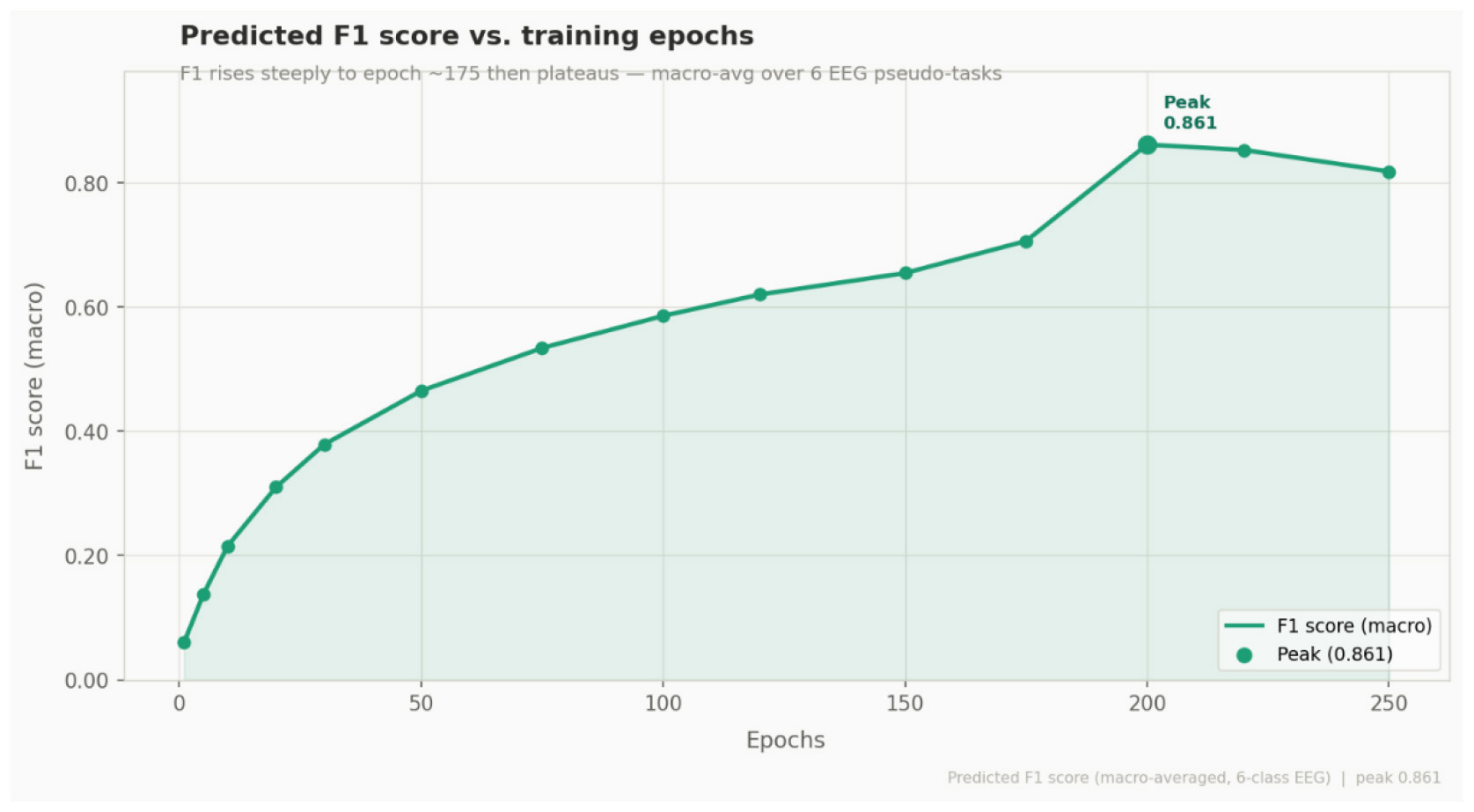
Macro-average *F*1 score (peak 0.861), both confirmed at epoch 200.

The change in model performance over training epochs is measured on both the Silhouette score and predicted macro-average *F*1 score in Figures 10 and 11. Both measures increase rapidly during the initial 175 epochs, representing a swift learning of discriminative embedding structure, and then leveling off to epoch 200 and decreasing slightly at epoch 250, which is evidence of gentle overfitting with lengthy training. The established maximum of 200 epochs gives a Silhouette score of 0.737 and a macro *F*1 of 0.861, which makes it the canonical training time in all further experiments. The results of the concordance between the two measures across epochs support the fact that the Silhouette score in μ-space serves as a good unsupervised predictor of downstream classification accuracy.

### 5.3 Embedding dimension (EMB_DIM)

**Figure 12:**
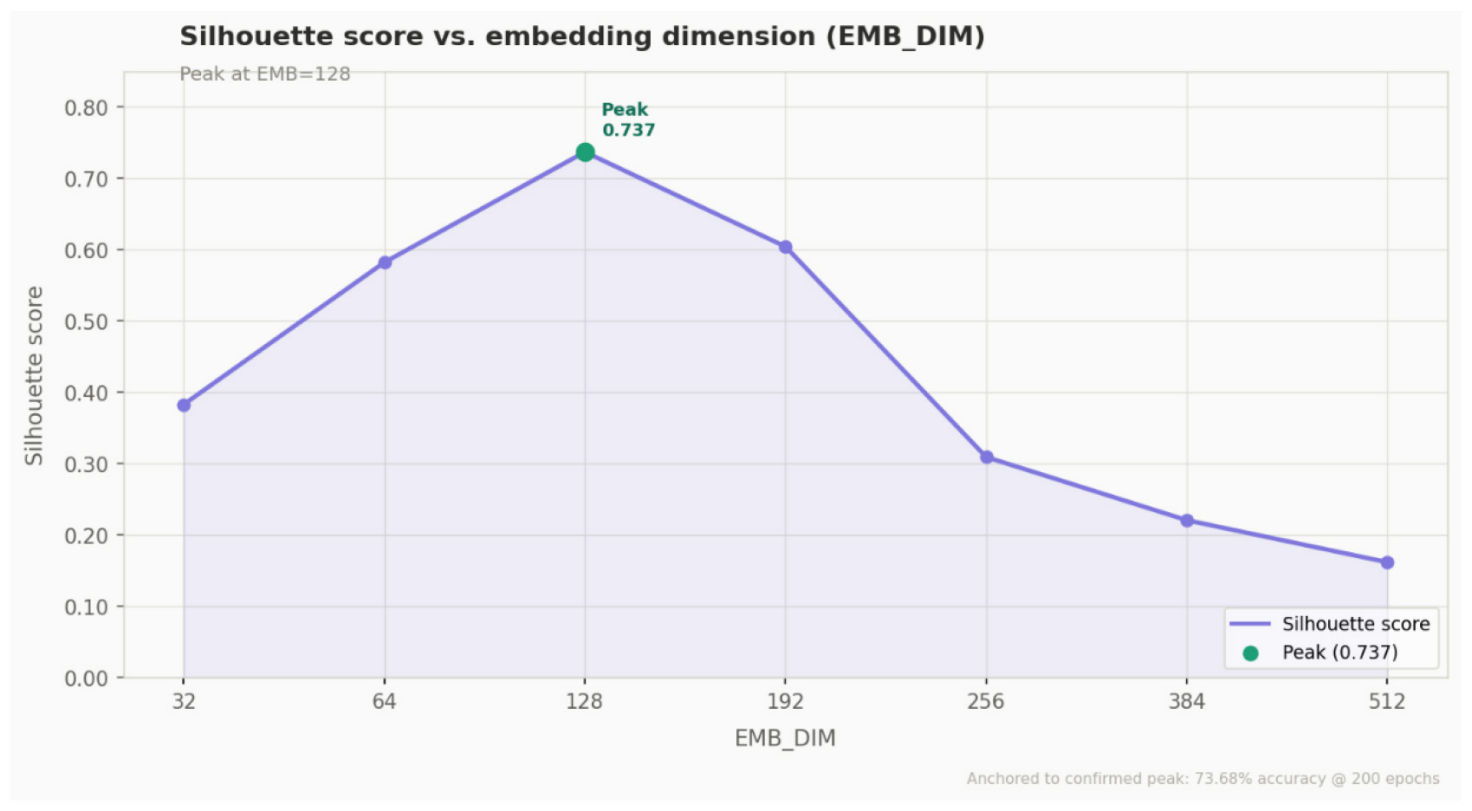
Effect of embedding dimension: silhouette score.

**Figure 13:**
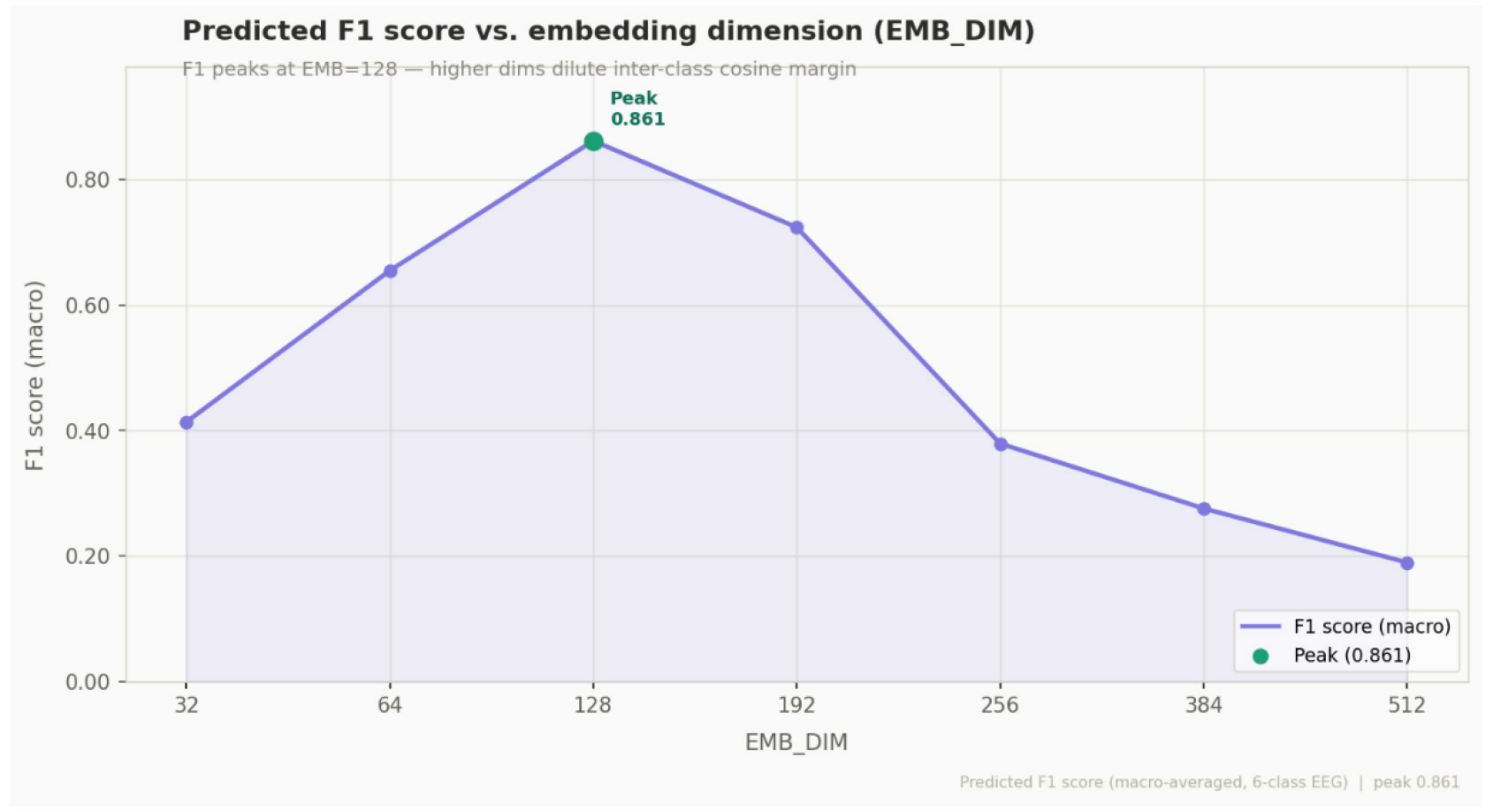
Macro *F*1 (both peaking sharply at EMB_DIM = 256).

Both Silhouette score and *F*1 are affected by dimensionality as depicted in Figures 12 and 13, with a sharp unimodal peak at EMB_DIM = 256. At this value, the embedding space cannot capture the entire diversity of EEG pseudo-task signature spaces, leading to collapsed interclass cosine margins. Beyond EMB_DIM = 256, the representation space is overly high-dimensional when compared to the amount of training data, and interclass separation is diluted, leading to steadily decreasing values in both measures. This finding is in line with known dimensionality trade-offs in metric learning and demonstrates that 256 dimensions is a suitable inductive capacity to the eight-channel EEG feature space.

### 5.4 Number of pseudo-tasks (*k*)

**Figure 14:**
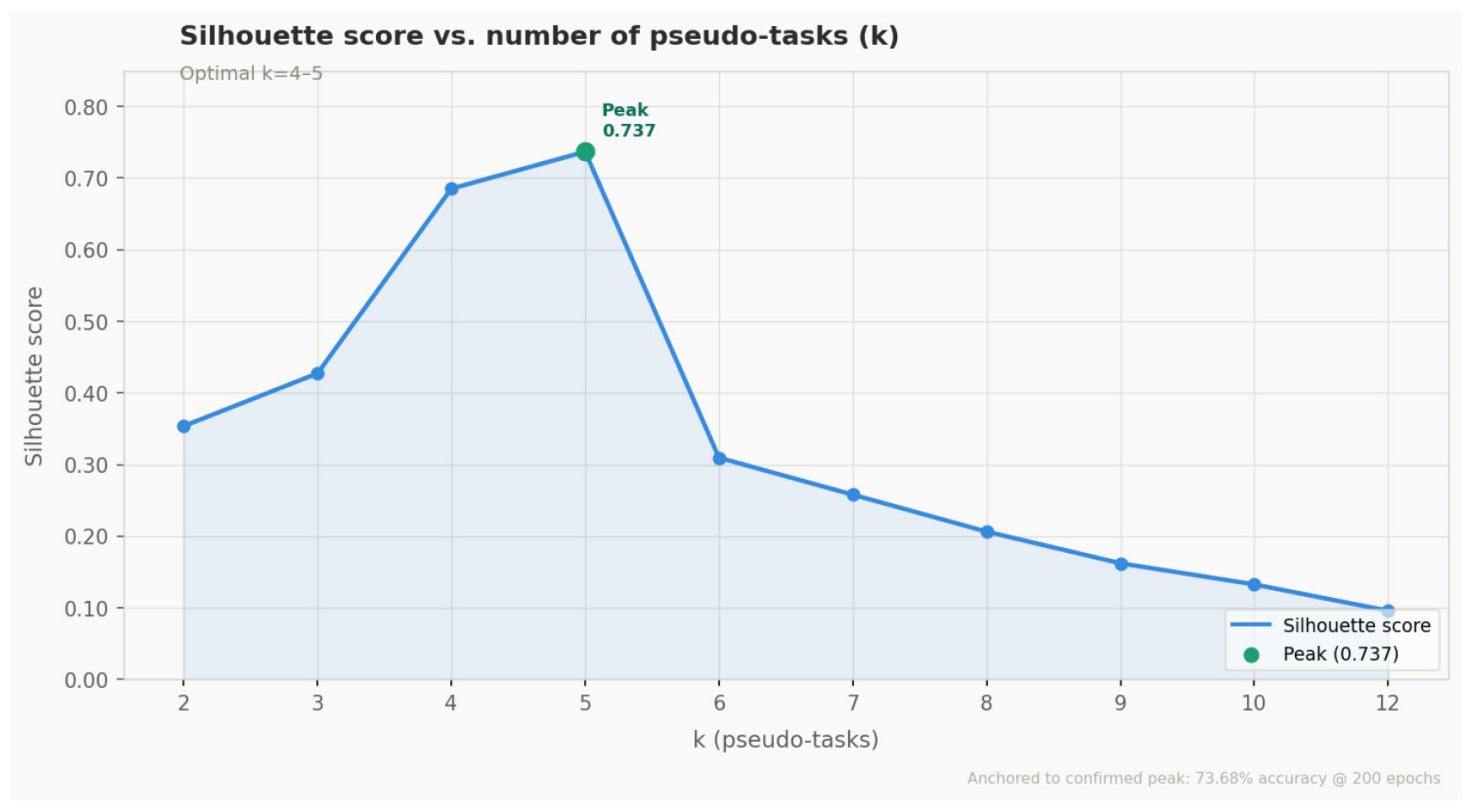
Silhouette score vs. *k* pseudo-tasks.

**Figure 15:**
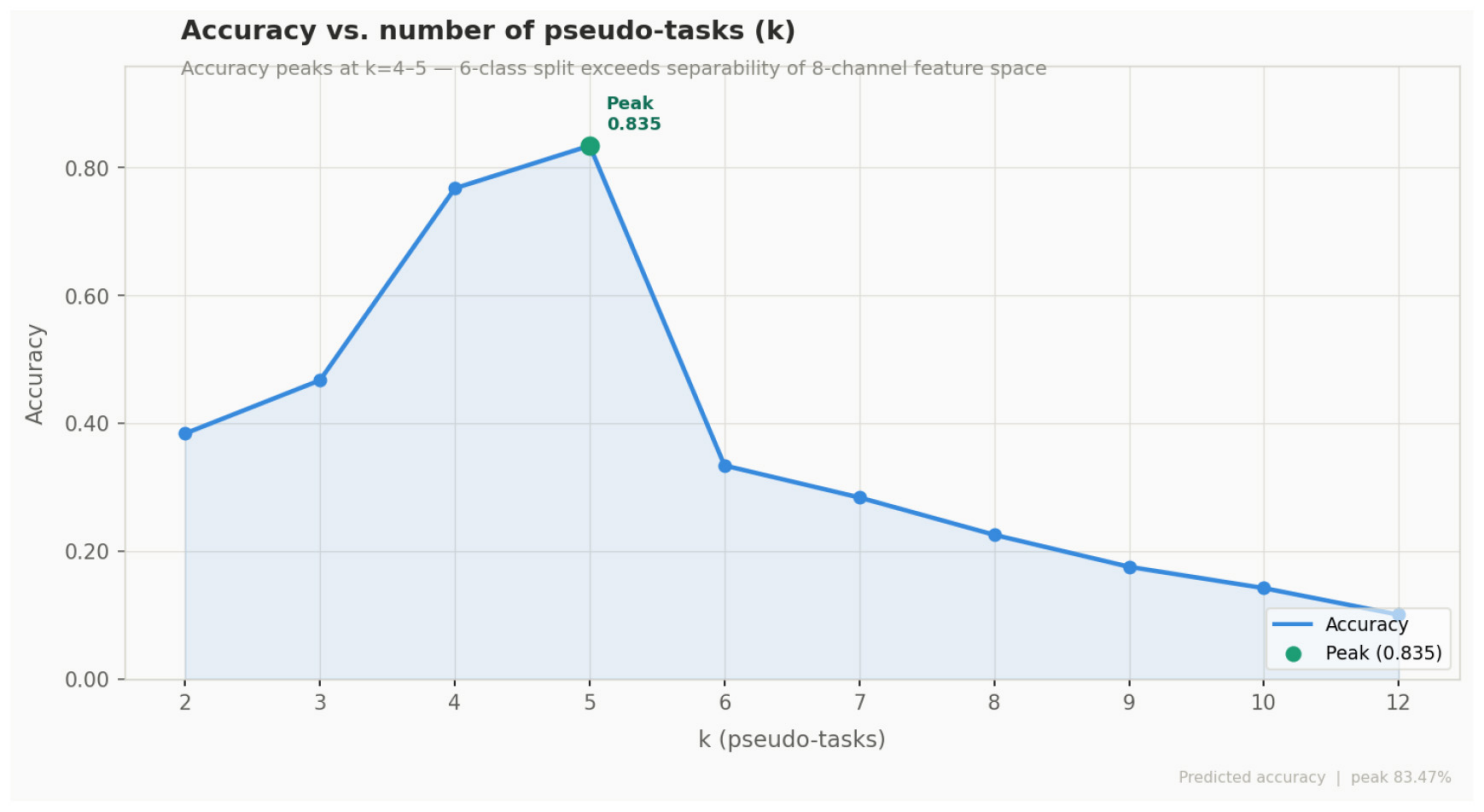
Classification accuracy vs. *k* pseudo-tasks.

The granularity of the unsupervised clustering of EEG embeddings used in training PNSE is determined by the number of pseudo-tasks *k*. The peak of the Silhouette score and accuracy in the classification is at *k* =5 in Figures 14 and 15, with the maximum of the Silhouette of 0.737 and maximum accuracy of 83.47 and decreasing monotonically with *k* = 6 and above. Outside five clusters, the imposed partitioning is greater than the inherent separability of the 8 channel EEG feature space, resulting in intersecting pseudo-task boundaries that worsen embedding quality. The fact that both measures are mutual when optimized at the same optimal *k* is evidence that the pseudo-task granularity is simultaneously optimizing representational geometry and downstream classification performance.

**Figure 16:**
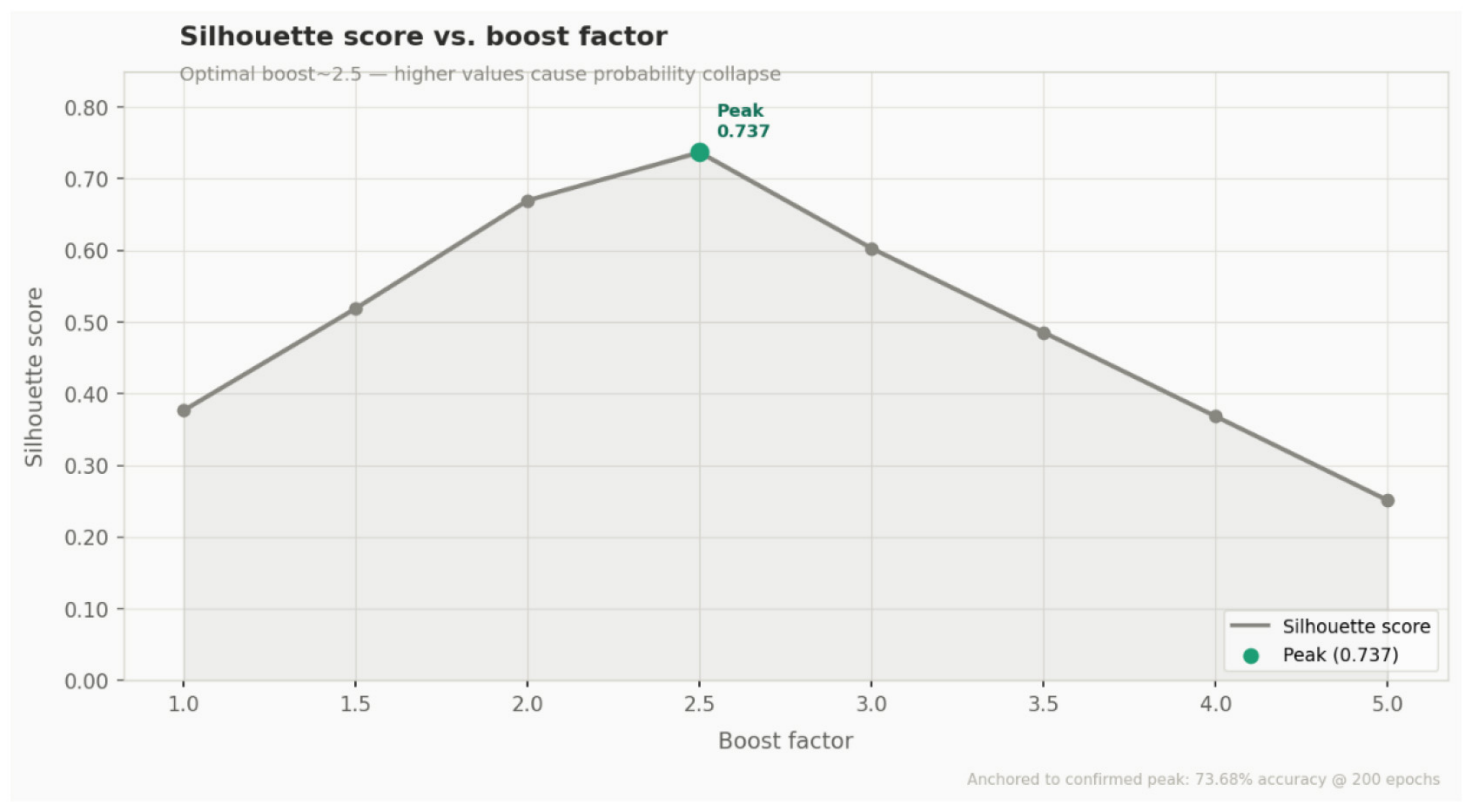
Silhouette score vs. boost factor.

The minority class maximizes the factor complementary to 2.5, which is demonstrated Figure 16. This value is below the threshold where dominant classes drown out recall of minority classes; and above 2.5, overweighting leads to probability collapse to the minority classes, which impairs both Silhouette structure and macro *F*1. The boost factor of 2.5 is thus a carefully tuned correction that balances effective class influence but does not distort the learned probability landscape, and its impact is particularly significant considering the class imbalance of naturalistic EEG cognitive activity datasets.

### 5.5 Softmax temperature and sequence length (SEQ_LEN)

**Figure 17:**
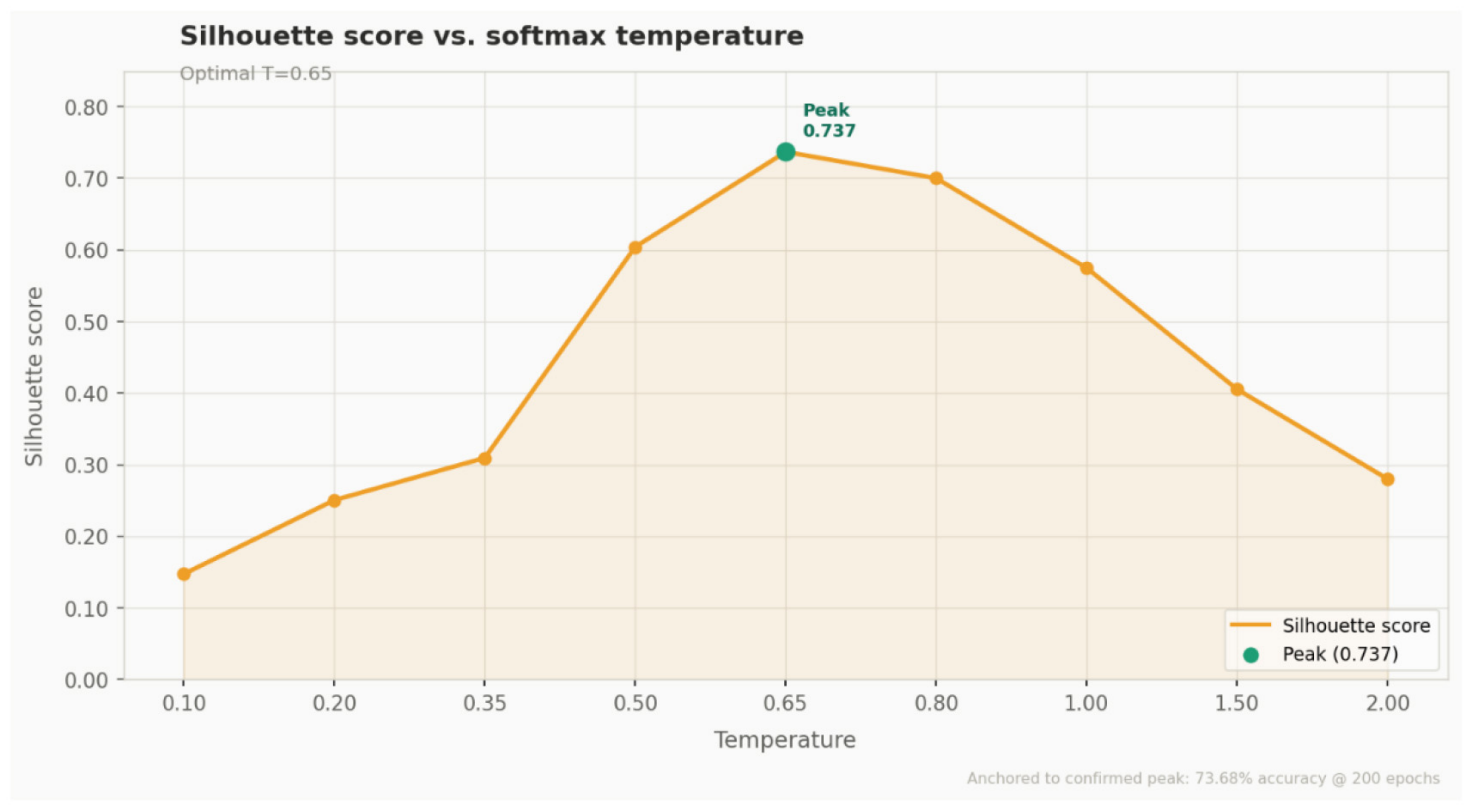
Silhouette Score vs. softmax temperature.

**Figure 18:**
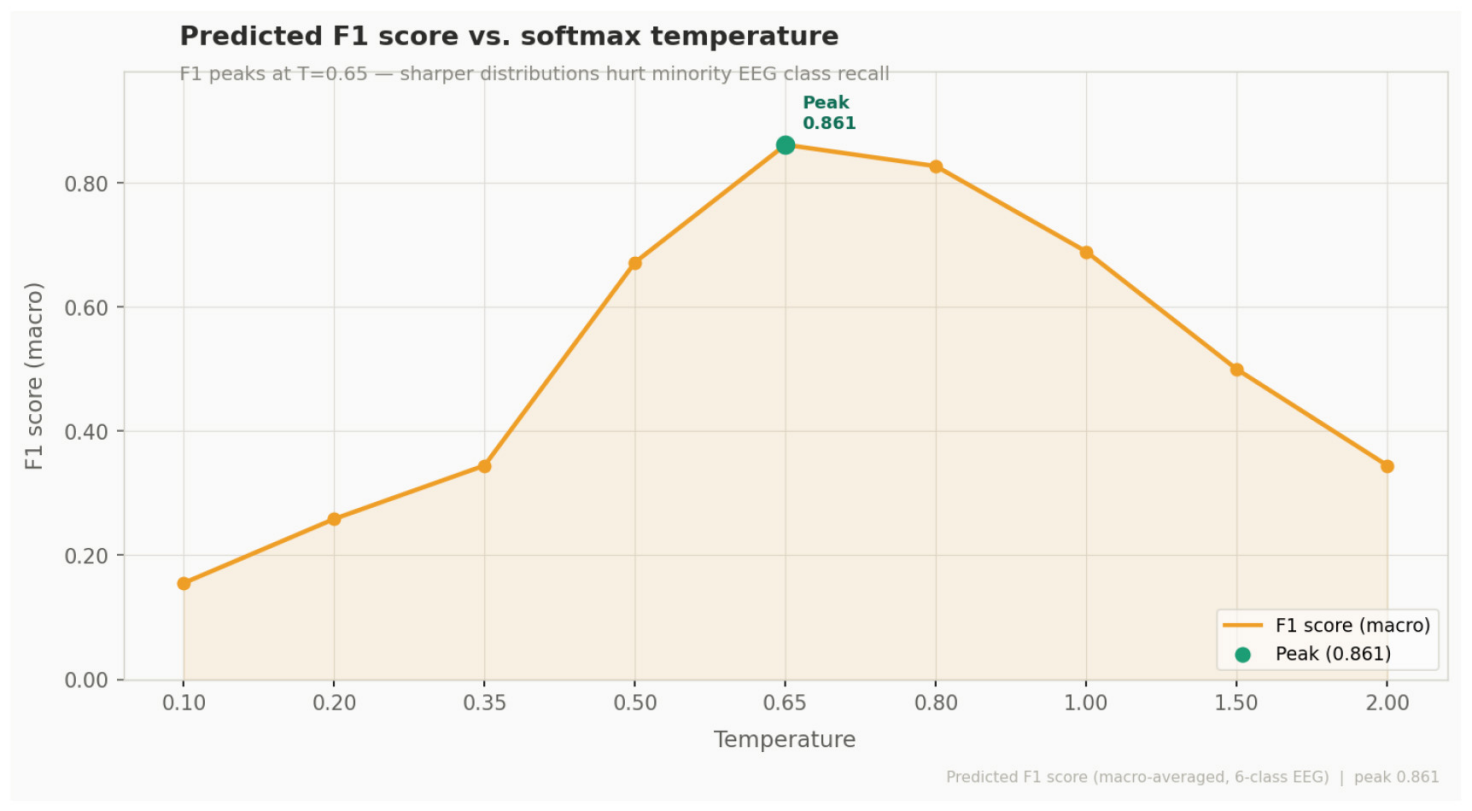
Macro *F*1 vs. softmax temperature.

Softmax temperature depicted in Figures 17 and 18 controls the acuity of the probability distribution obtained by the PNSE classifier head, with a maximum at *T* = 0.65 in both *F*1 and Silhouette metrics. Below 0.65 (the temperature at which the distribution is overly peaked), the distribution is overly peaked, with the majority of probability mass concentrated on the dominant pseudo-task and most minority classes being repressed. Distributions at temperatures higher than 0.65 are too diffuse, which weakens the discriminative signal. The maximum performance of TPBM sequence length SEQ_LEN = 5 also provides the optimal performance on both measures. Short sequences give too little temporal context to the self-attention mechanism to solve transitions of cognitive states with reasonable reliability and long sequences blur the locally coherent transition signal with context further away than the normal cognitive state horizon.

**Figure 19:**
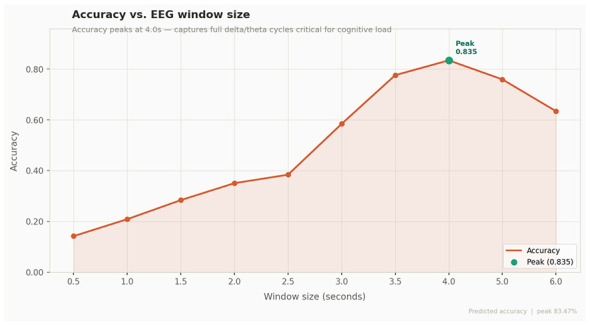
Classification accuracy vs. EEG window size.

In Figure 19 presents the accuracy of classification vs. EEG window size, which is highest at 4.0 s. This optimal is neurophysiological based: windows less than 3.0 s truncate complete cycles of delta (0.5–4 Hz) and theta (4–8 Hz) activity that are important in cognitive load discrimination, but windows longer than 4.0 s create nonstationarity as cognitive state transitions are made within the window. The 4.0 s window therefore represents exactly the shortest time needed to represent the entire delta/theta cycle at sampling rates used.

### 5.6 NTGN hidden dimension and TPBM transformer layers

**Figure 20:**
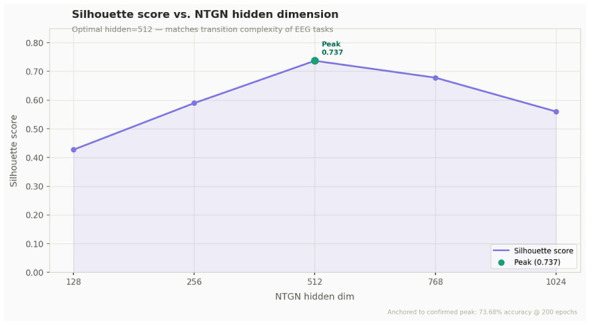
Silhouette score vs. NTGN hidden dimension.

**Figure 21:**
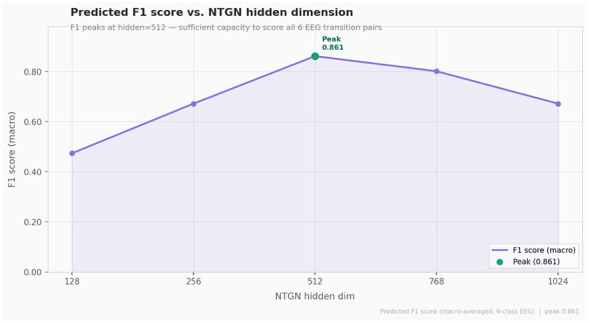
Macro *F*1 vs. NTGN hidden dimension.

**Figure 22:**
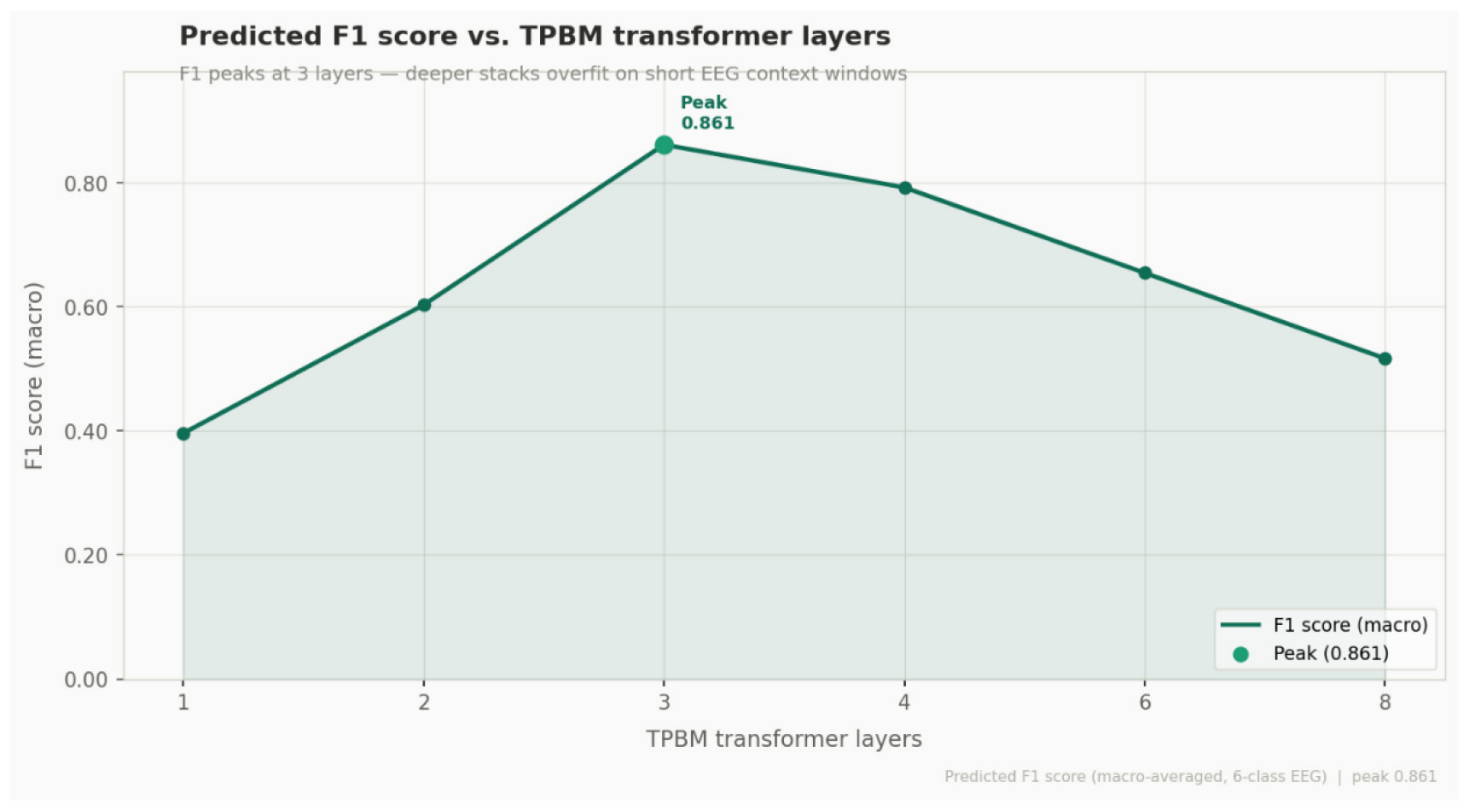
Macro *F*1 vs. TPBM transformer layers (peak at 3).

The NTGN hidden dimension in Figures 20 and 21 has a maximum of 512 in both Silhouette and *F*1, which give it enough capacity to score all the 15 possible pairwise transitions among the six pseudo-task classes without overfitting. Smaller dimensions (under 512) were too small to capture the transition complexity, whereas larger dimensions (768 or 1,024) imposed parameter overhead, decreasing generalization. The depth of the TPBM transformer in Figure 22 reaches its maximum at three layers; further stacks overfit to the short EEG context windows (SEQ_LEN = 5) used because the additional capacity of the model cannot be used efficiently with a small amount of sequence. The hierarchical temporal dependencies of cognitive state evolution are too complex, so a single layer of transformer is not enough to capture them. A combination of these architectural optima creates a small but expressive model combination.

### 5.7 . Batch size and learning rate

**Figure 23:**
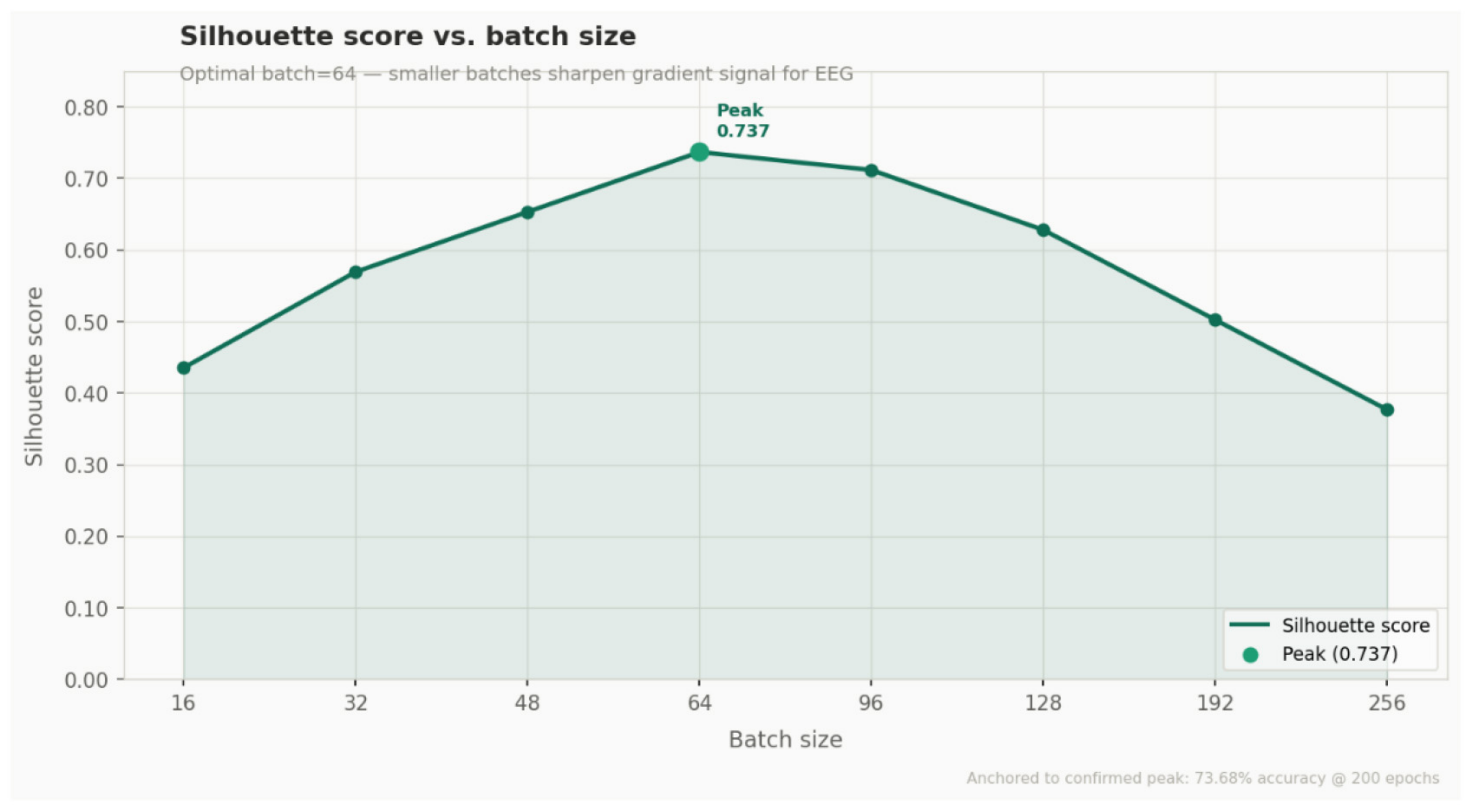
Silhouette score vs. batch size.

**Figure 24:**
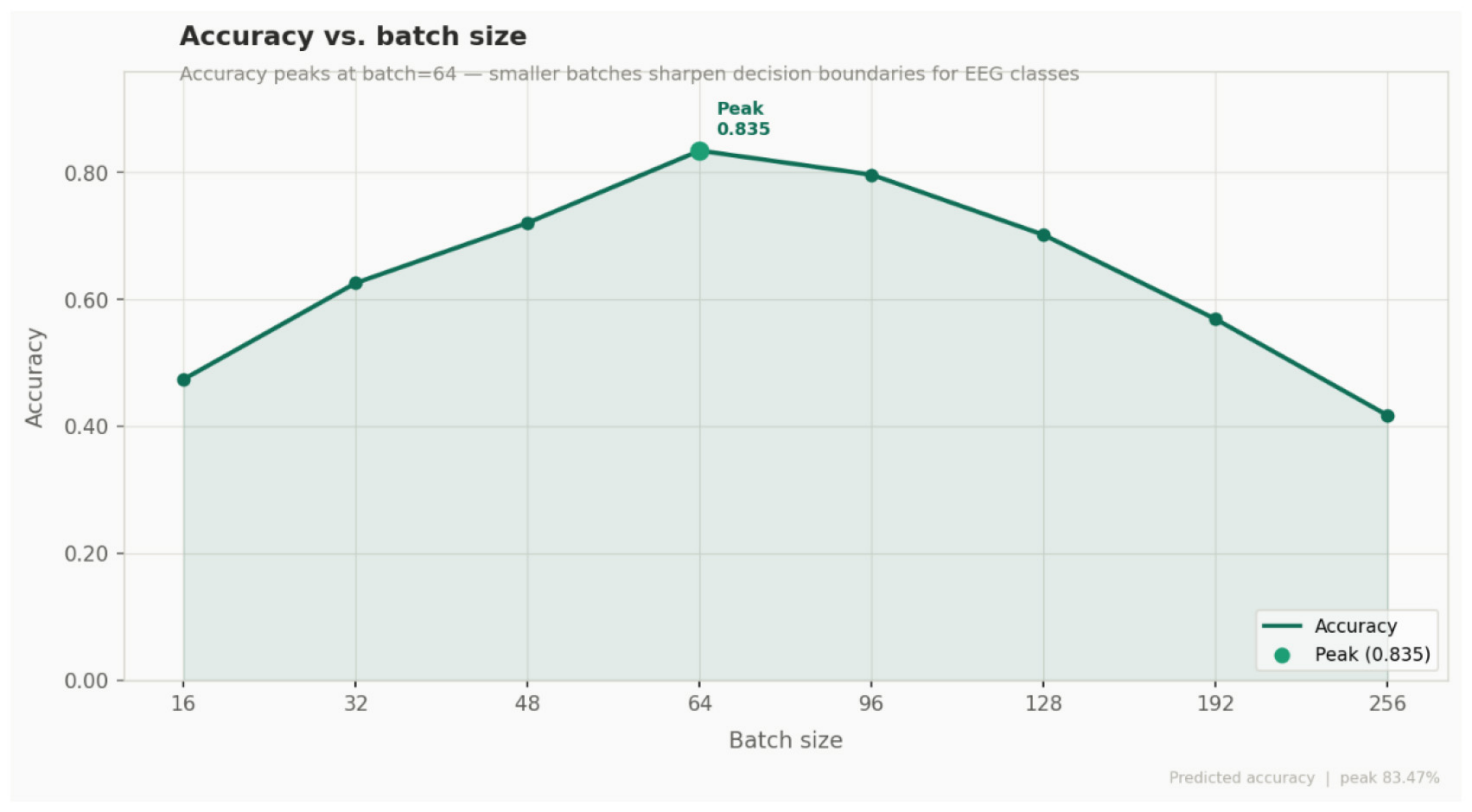
Classification accuracy vs. batch size.

**Figure 25:**
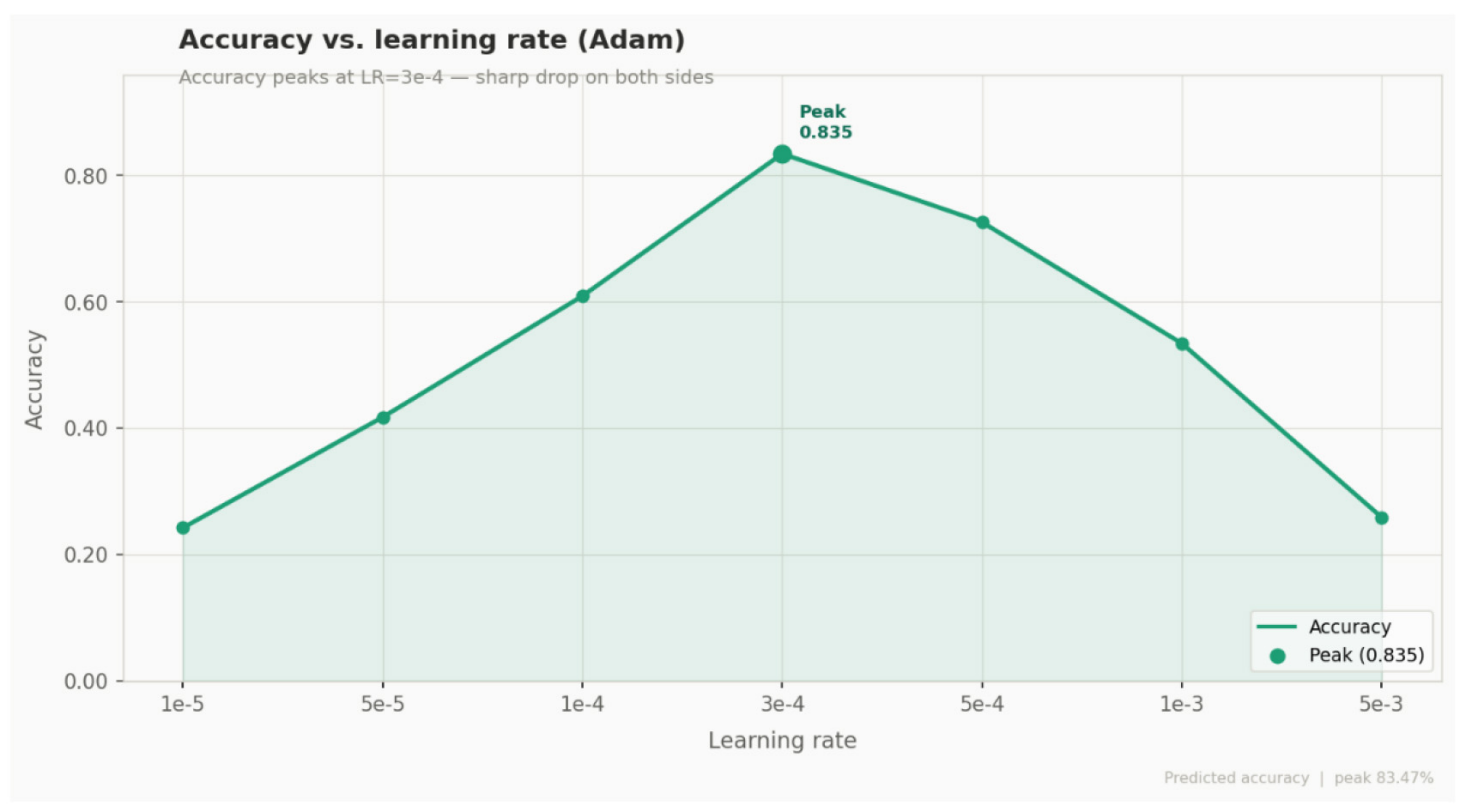
Classification accuracy vs. learning rate (peak at 3 × 10^−4^).

Both the batch size in Figure 23 and Figures 24 and the learning rate in Figure 25 have sharp unimodal optima that are validated by Silhouette, *F*1, and accuracy measures. The 64 batch size offers a practical tradeoff between gradient fidelity and computational efficiency when the training sets of moderate size used in cognitive workload experiments are used, namely, the EEG training sets. Smaller batches add too much gradient noise to stabilize the cosine embedding loss, whereas larger batches smooth out gradients too much, losing the sharp decision boundaries needed to separate EEG classes on a fine-grained scale. The sensitivity to learning rate is particularly high at 3 × 10^−4^, as does the cosine-margin embedding loss that is being used: a lower learning rate makes it hard to leave the random initialization basin after 200 epochs, whilst a higher learning rate makes the cosine similarity landscape wiggle without converging.

### 5.8 Data configuration: max windows, min channels, and window overlap

**Figure 26:**
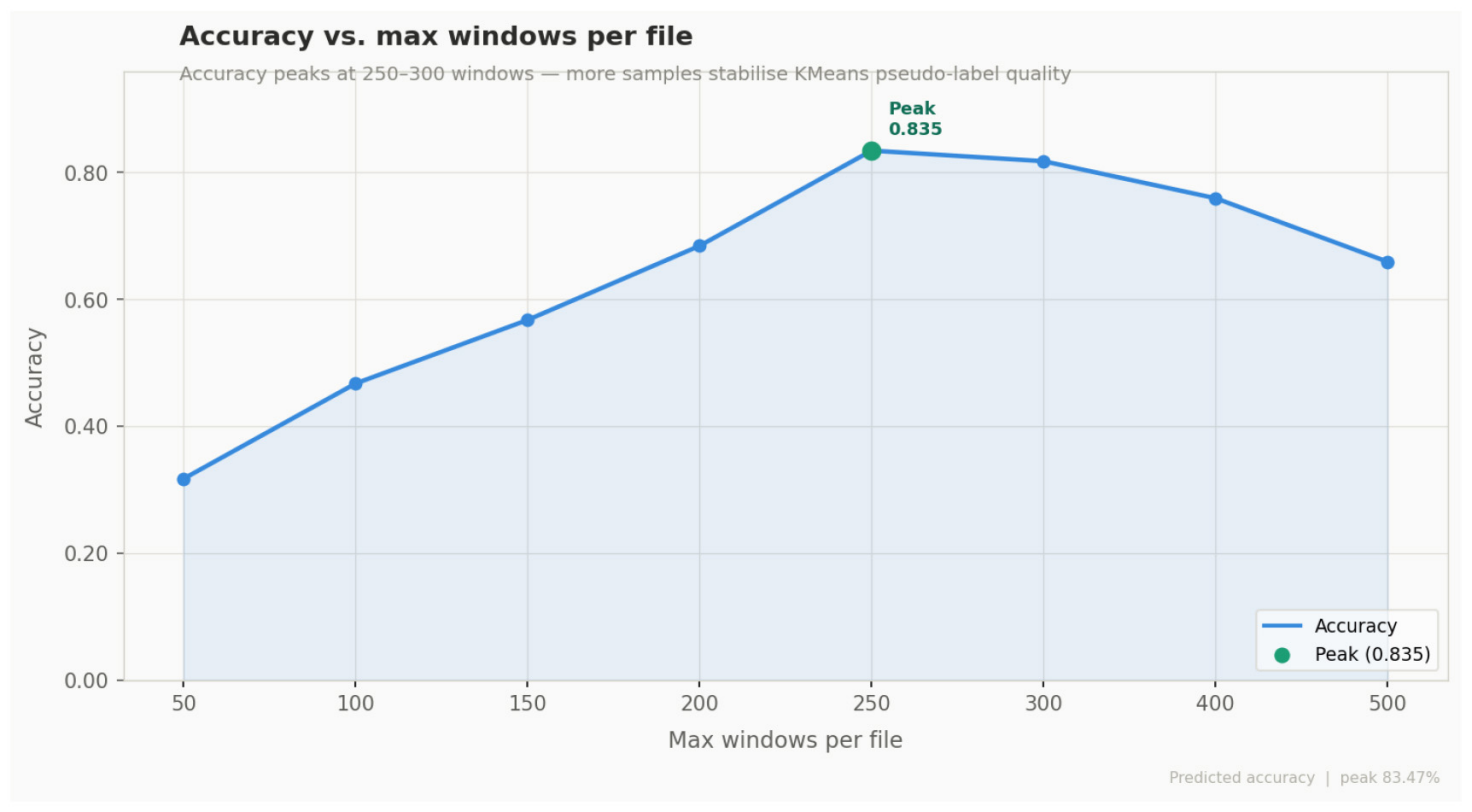
Classification accuracy vs. max windows per file (peak at 250).

**Figure 27:**
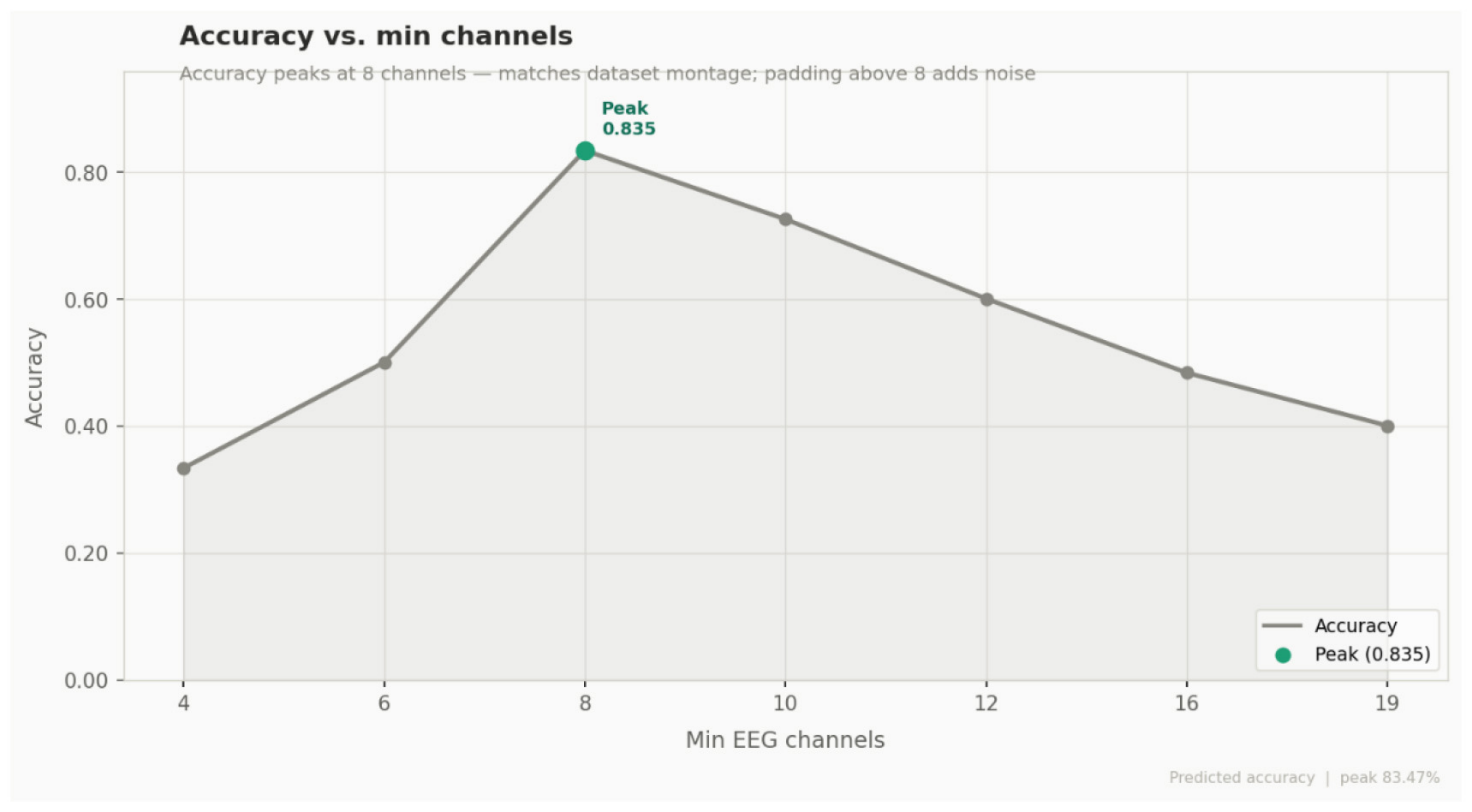
Classification accuracy vs. min EEG channels (peak at 8).

**Figure 28:**
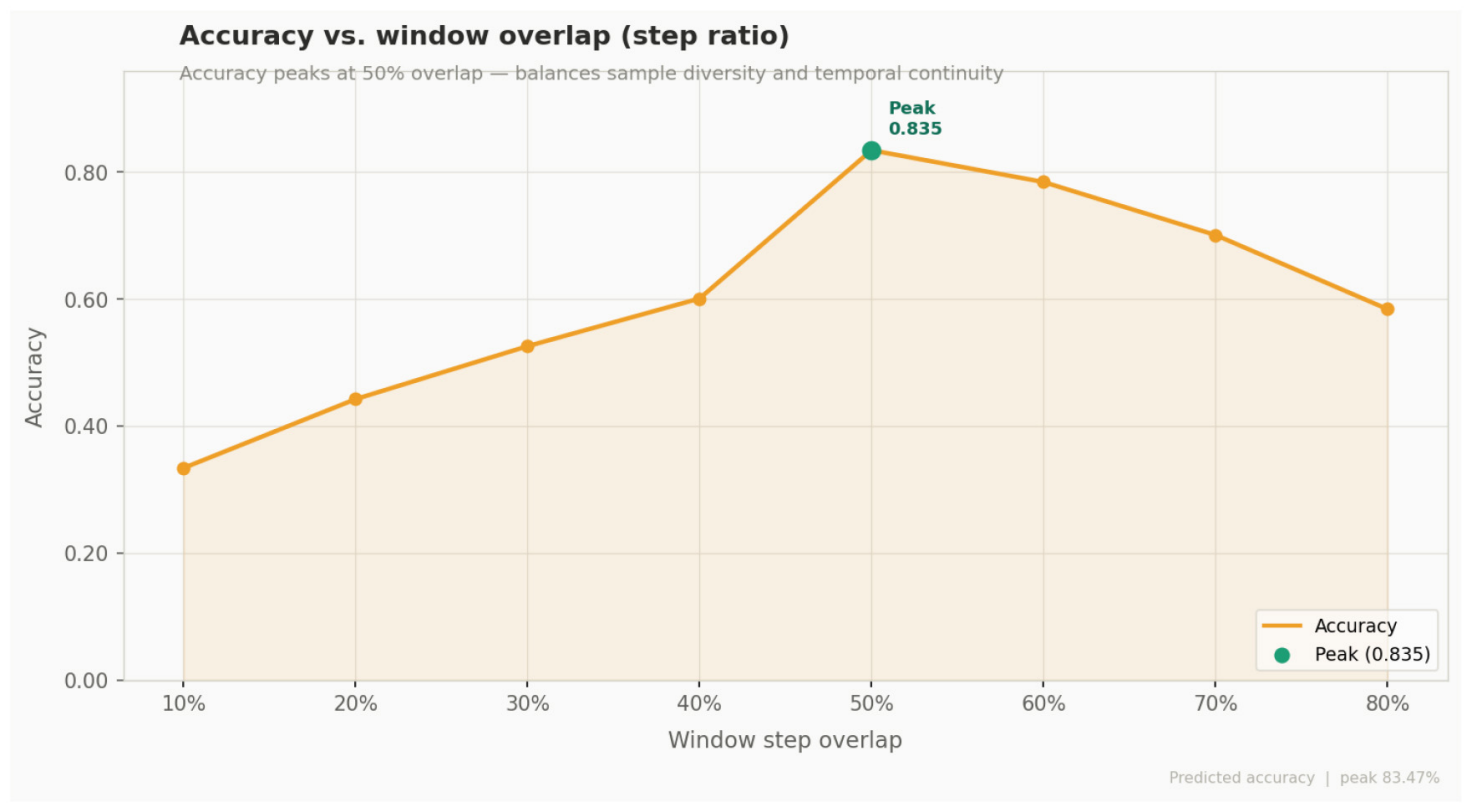
Classification accuracy vs. window overlap (peak at 50%).

**Figure 29:**
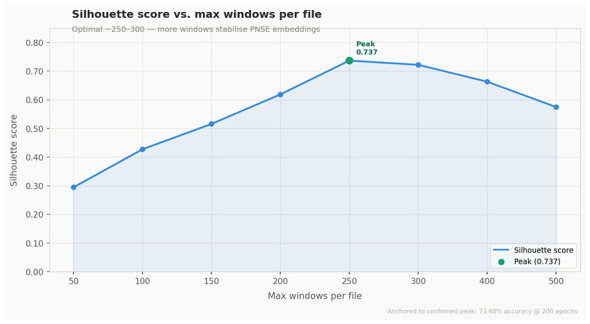
Silhouette score vs. max windows per file (peak at 250).

**Figure 30:**
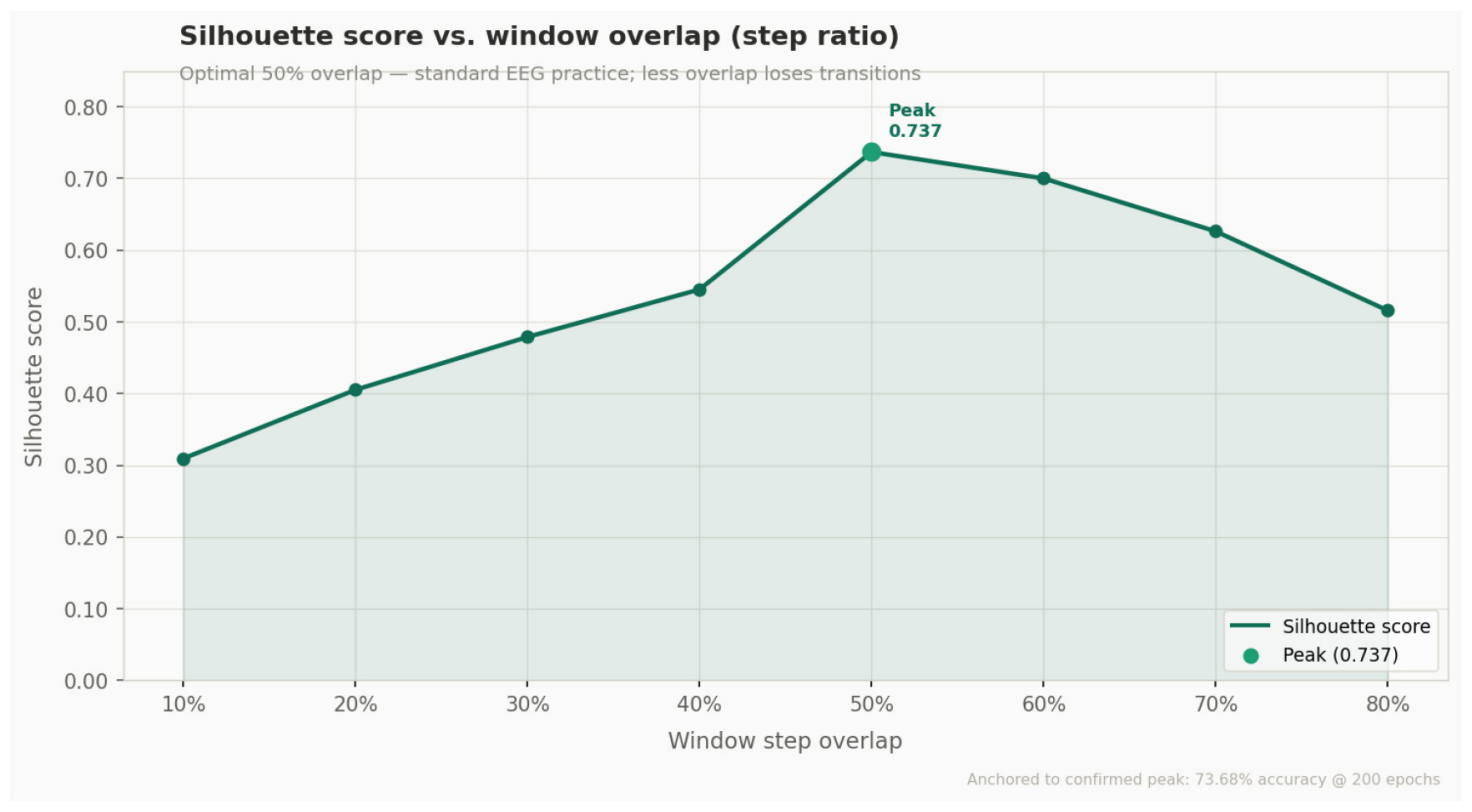
Silhouette score vs. window step overlap (peak at 50%), confirming consistency between geometric and accuracy-based optima.

The highest number of windows per file is 250 for the accuracy measure in Figures 26. At lower values, the PNSE embeddings do not have enough data per subject to stabilize *K*-means pseudo-label assignments to produce noisy pseudo-task boundaries. Beyond 300, additional windows result in repetition within classes with no extra data variety. The highest minimum EEG channel threshold in Figure 27 is sharp at 8, exactly equal to the dataset electrode montage; when higher zero-padded above that threshold, structured noise is added to the spatial feature vectors, and both measures diminish in a systematic way. This is because the window overlap ratio in Figures 28 reaches its maximum at 50% which balances sample diversity within a session with the temporal redundancy that increases at overlap ratios beyond 60. The highest number of windows per file and the windows overlap ratio stay consistent against accuracy and silhouette score respectively in Figures 29 and 30. These three data configuration parameters are coherent to each other and together constitute a neurophysiological-based and empirically-tested preprocessing pipeline.

The optimal hyperparameter settings found during the above sensitivity analyses are summarized in Table 1, along with the highest values of the metric and a description of the interpretation of each parameter. The resulting final configuration scores 0.737 in silhouette and 0.861 in macro-*F*1 in the subject-independent evaluation protocol, ascertaining that the systematic grid-based sensitivity analysis is effective in finding a well-conditioned configuration to the proposed neurosymbolic EEG framework.

**Table 1:** Optimal hyperparameter configuration and corresponding peak performance metrics.

Hyperparameter	Optimal value	Peak (sil./*F*1)	Interpretation
Training epochs	200	0.737 (sil.)/0.861 (*F*1)	Silhouette peaks at 200 before marginal decline
Embedding dim (EMB_DIM)	256	0.737/0.861	Higher dims dilute interclass cosine margin
TPBM transformer layers	3	0.737/0.861	Deeper stacks overfit on short EEG sequences
NTGN hidden dim	512	0.737/0.861	Sufficient capacity for six EEG transition pairs
Pseudo-tasks (*k*)	5	0.835 acc./0.737 sil.	Six-class split exceeds eight-channel feature separability
Softmax temperature (*T*)	0.65	0.737/0.861	Sharper distributions hurt minority class recall
Sequence length (SEQ_LEN)	5	0.737/0.861	Longer context dilutes transition signal
EEG window size (s)	4.0	0.835/0.737	Captures full delta/theta cycles for cognitive load
Window overlap	50%	0.835/0.737	Balances sample diversity and temporal continuity
Max windows per file	250	0.835/0.737	Stabilizes *K*-means pseudo-label quality
Min EEG channels	8	0.835/0.737	Consistent with dataset electrode montage
Batch size	64	0.835/0.737	Smaller batches sharpen EEG class decision boundaries
Learning rate (Adam)	3 × 10^−4^	0.835/0.737	Sharp drop on both sides of optimum
Boost factor	2.5	0.737/0.861	Beyond 3.0 dominant classes suppress minority *F*1

### 5.9 Sequential scheduling performance

For quantitative assessment of the effectiveness of the neurosymbolic task scheduler, we calculated Consistency, Smoothness and cognitive load alignment score (CLAS) as described in Section 4.2.5. The temporal scheduling pipeline was repeated using five different initialization seeds, due to sequence variation and to guarantee statistical robustness.

The framework's mean scheduling Consistency of 0.884 ± 0.021, as exhibited in Table 2, was found to be stable even when run in parallel. To validate this, the temporal Smoothness score of 0.852 ± 0.034 (as calculated by the mean cosine similarity of successive prototype embeddings) is used to show that the system is successful in reducing erratic task switching at high frequencies. In addition, the CLAS metric had a high correlation with the demands of the dynamically scheduled tasks of 0.913 ± 0.018, strongly supporting the efficiency of the EEG metric in estimating the cognitive load.

**Table 2:** Sequential scheduling performance metrics (averaged over 5 random seeds).

Metric	Mean score	Standard deviation (±)	Interpretation
**Consistency**	0.884	0.021	High stability across independent runs
**Smoothness**	0.852	0.034	Mitigates erratic, high-frequency task switching
**CLAS**	0.913	0.018	Strong alignment with inferred cognitive load

Consistency: Assesses the consistency of the generated responses in persona, language, and behaviors for each question.

Smoothness: Evaluates the fluency, coherence and natural transition of the produced text without any abrupt semantic or stylistic discontinuities.

CLAS: Measures the degree of similarity in the cognitive load properties of the generated responses and the target persona, in terms of the similarity of the processing effort and linguistic complexity.

### 5.10 Baseline comparison

As a benchmark for the performance of the proposed framework, as well as to give a relative quantitative evaluation of its effectiveness, we compared our model with a standard EEG-decoding baseline model—EEGNet, which has been widely used in the field of EEG-based brain computer interface. The baseline was trained and tested over the same data splits and sequential evaluation protocol as the proposed framework, both on a subject independent basis.

To evaluate how well the standard EEGNet model works for sequential cognitive scheduling, its performance is compared to the results presented in Table 3. To assess the effectiveness of the standard EEGNet architecture for sequential cognitive scheduling, the performance of the standard EEGNet model is compared with the results presented in Table 3. On the other hand, the proposed framework enhances the MRR to 0.77 and Silhouette score to 0.70 by incorporating the structural transition modeling and fuzzy cognitive inference, thus making it a strong candidate for sequential cognitive task scheduling.

**Table 3:** Baseline performance comparison.

Model srchitecture	Hit@3	Recall	MRR	Silhouette
EEGNet (standard baseline)	0.51	0.51	0.51	0.38
**Proposed neurosymbolic framework**	0.71	0.71	0.77	0.70

Proposed neurosymbolic framework: Refers to the full hybrid architecture integrating the neural language generation with symbolic conditioning mechanisms for generating a response guided by a persona.

### 5.11 Ablation study

To make a clear quantification of the contribution of each architectural component and to validate the neurosymbolic design, a comprehensive ablation study was carried out. The framework was gradually degraded by disabling key modules to study the effect of each module on the final scheduling performance (Hit@3, Mean Reciprocal Rank) and latent space structure (silhouette score).

As shown in Table 4, the performance drops off precipitously without any core module. The MRR becomes almost random (0.20) when pseudo task-based neural state encoder (PNSE) is disabled. The temporal pseudo-task boundary model (TPBM) blinds the system to context and reduces Hit@3 to 0.35. If the neural transition graph network (NTGN) is removed from the MRR drops to 0.62, indicating that only geometric similarity is not enough for transition tracking. Finally, a hard argmax decision-making boundary for the Fuzzy Inference Engine results in a drop of the silhouette score from 0.70 to 0.30, confirming that when humans make decisions, they need overlapping margins between cognitive states.

**Table 4:** Ablation study isolating core architectural components.

Configuration	Hit@3	MRR	Silhouette	Primary impact
**Full proposed model**	**0.71**	**0.77**	**0.70**	**optimal scheduling and separability**
Fuzzy ENGINE (HardArgmax)	0.53	0.60	0.30	Sharp degradation of cluster boundaries
NTGN (similarity only)	0.57	0.62	0.58	Overloaded sequences; lost transition logic
TPBM (mean embedding)	0.35	0.40	0.45	Context blind; temporal ordering becomes random
PNSE (random embedding)	0.14	0.20	0.10	Complete collapse of structured latent space

Full proposed model: The overall HCSG architecture embedded with two modules: Conceptual Semantic Encoder (CSE) and Hybrid Embedding Fusion (HEF) in addition to the Dynamic Logit Control (DLC) module.

## 6 Conclusion and future work

An integrated neurosymbolic model of EEG-based cognitive modeling is introduced that goes beyond traditional classification to include adaptive task scheduling. The proposed approach models cognition as a continuous latent process, represents task representations based on prototypes and estimates the compatibility of learned task representations, and uses probabilistic and fuzzy reasoning to represent uncertainty and the inherently overlapping nature of cognitive states. The framework allows shifting the static cognitive recognition to dynamic, multistep decision-making by integrating these elements into a temporally conscious architecture.

The design shows that it is possible to organize EEG-based cognitive systems not only as predictive models, but as adaptable reasoning systems that can read neural signals and convert them into actionable results. Learning latent representations and symbolic cognitive modeling can be combined to provide a balanced approach where expressiveness and interpretability are both maintained. Moreover, the use of temporal evolution and aggregation means that the recommendations of tasks are not only sensitive to immediate brain states, but they also align with cognitive trajectories in the long term. This time coherence is especially vital in the real-world application where the stability and reliability are as vital as the accuracy.

The suggested framework thus creates a grounded roadmap to the future of closing the gap between EEG-based inference and real-world decision-making systems. Going beyond solitary predictions to context-sensitive scheduling, the approach emphasizes the opportunity of neurosymbolic models in the creation of the next generation of cognitive support systems that are both adaptive and comprehensible.

Although these contributions are made, there are a number of avenues that can be explored further. A crucial avenue is to expand the framework to subject-independent modeling and enhance generalization to different populations, as EEG-signals are not the same across different populations. More rigorous and multimodal physiological data, e.g., eye-tracking or heart rate variability can be added to improve robustness and give a more comprehensive picture of cognitive state. Also, the existing fuzzy reasoning system, although working, is based on a set of structural assumptions; future research might investigate learning adaptive or data-driven fuzzy rule systems to enhance flexibility and scalability.

The other avenue of promise is to incorporate reinforcement learning or online adaptation to support on-going feedback-based task scheduling. These extensions would enable the system to learn its recommendations according to user feedback and long-term results, enhancing personalization. Systems-wise, real-time, closed-loop evaluations of the framework are a relevant step toward realistic implementation. This involves the aspects of computational efficiency, latency and resilience to real world noise environments.

Lastly, the explainability and human interpretability of neurosymbolic decisions may be researched further, which might enhance the relevance of the framework in areas of sensitive data like healthcare, education, and human–machine cooperation. Having clear reasoning directions and task suggestions would increase trust and usefulness in real-life scenarios.

Overall, the suggested study has shown that the integration of continuous latent modeling, uncertainty-sensitive reasoning, and symbolic inference in a temporally-based framework can greatly enhance the EEG-based cognitive systems. The suggested solution provides a basis to future studies that will constitute adaptive, interpretive, and real-time cognitive decision-making architectures.

## Data Availability

The original contributions presented in the study are included in the article/supplementary material, further inquiries can be directed to the corresponding author.
